# Bioinspired Passive Tactile Sensors Enabled by Reversible Polarization of Conjugated Polymers

**DOI:** 10.1007/s40820-024-01532-z

**Published:** 2024-09-27

**Authors:** Feng He, Sitong Chen, Ruili Zhou, Hanyu Diao, Yangyang Han, Xiaodong Wu

**Affiliations:** 1 School of Mechanical Engineering, Sichuan University, Chengdu, 610065 People’s Republic of China; 2 School of Software Engineering, Chongqing University of Posts and Telecommunications, Chongqing, 400065 People’s Republic of China; 3 State Key Laboratory of Polymer Materials Engineering, Sichuan University, Chengdu, 610065 People’s Republic of China

**Keywords:** Passive tactile sensors, Reversible polarization of conjugated polymers, Tactile perception, Machine learning algorithm, Object recognition

## Abstract

Tactile perception plays a vital role for the human body and is also highly desired for smart prosthesis and advanced robots. Compared to active sensing devices, passive piezoelectric and triboelectric tactile sensors consume less power, but lack the capability to resolve static stimuli. Here, we address this issue by utilizing the unique polarization chemistry of conjugated polymers for the first time and propose a new type of bioinspired, passive, and bio-friendly tactile sensors for resolving both static and dynamic stimuli. Specifically, to emulate the polarization process of natural sensory cells, conjugated polymers (including poly(3,4-ethylenedioxythiophene):poly(styrenesulfonate), polyaniline, or polypyrrole) are controllably polarized into two opposite states to create artificial potential differences. The controllable and reversible polarization process of the conjugated polymers is fully in situ characterized. Then, a micro-structured ionic electrolyte is employed to imitate the natural ion channels and to encode external touch stimulations into the variation in potential difference outputs. Compared with the currently existing tactile sensing devices, the developed tactile sensors feature distinct characteristics including fully organic composition, high sensitivity (up to 773 mV N^−1^), ultralow power consumption (nW), as well as superior bio-friendliness. As demonstrations, both single point tactile perception (surface texture perception and material property perception) and two-dimensional tactile recognitions (shape or profile perception) with high accuracy are successfully realized using self-defined machine learning algorithms. This tactile sensing concept innovation based on the polarization chemistry of conjugated polymers opens up a new path to create robotic tactile sensors and prosthetic electronic skins.

**Supplementary Information:**

The online version contains supplementary material available at 10.1007/s40820-024-01532-z.

## Highlights

- Fully organic and passive tactile sensors are developed via mimicking the sensing behavior of natural sensory cells.
- Controllable polarizability of conjugated polymers is adopted for the first time to construct passive tactile sensors.
- Machine learning-assisted surface texture detection, material property recognition, as well as shape/profile perception are realized with the tactile sensors.

**Supplementary Information:**

The online version contains supplementary material available at 10.1007/s40820-024-01532-z.

## Graphical abstract

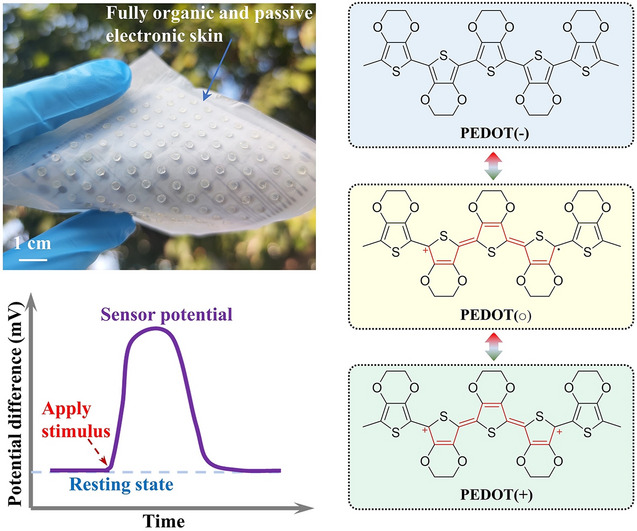

## Introduction

Tactile sensation, a fundamental sense of the human body, enables us to acquire a wide spectrum of information that we leverage for object recognition and manipulation, including pressure, shear, strain, vibration, sliding, and so on [1, 2]. Tactile sensation is also a crucial medium for us to safely and efficiently navigate our surrounding environment. Imitating the tactile sensing functions in natural skin has attracted enormous attention in the emerging fields of advanced robots and prosthesis, biomedical equipment, and human-interactive systems [3–6].

In the past decades, artificial tactile sensors have witnessed remarkable progress, marked by advancements in high sensitivity [7, 8], low detection limit [9], multiple functionalities [10, 11], self-healing capability [12, 13], high pixel density [14], etc. [15]. Looking into these achievements, most of them are realized based on active sensing principles, including resistive sensing [16], capacitive sensing [17], transistor-based sensing [7], optical sensing [18], magnetic sensing [19], and so on [20]. These active sensing devices are operated by applying a source signal (e.g., current and voltage) to the devices first and then measuring specific electrical parameters (e.g., resistance, capacitance, and current) that are encoded with tactile information. Active tactile sensors are capable of detecting static and dynamic tactile stimulations, but they require a continuous power supply even in a standby state, resulting in high power consumption up to milli-watts per sensing unit. Such high power consumption limits their practical applications, especially for the isolated tactile sensing systems without external power supply.

In contrast, passive sensing devices (such as piezoelectric, triboelectric, and piezoionic devices) do not necessitate external energy supply and can generate signal outputs by themselves when subjected to tactile stimuli, featuring ultralow power consumption [21–24]. Nevertheless, these passive tactile sensors typically respond only to dynamic or transient mechanical stimulations, unable to resolving static and slowly varying tactile stimuli, restricting their application scenarios in comprehensive tactile analysis. Recently, a passive electrochemical sensing mechanism is proposed to resolve static touch stimulations [25–29]. However, these sensing devices necessitate sophisticated organic/inorganic hybrid material systems (e.g., Ag/AgCl-Fe^2+^/Fe^3+^, and VO_2_/MnHCF/MoS_2_-Zn/Zn^2+^), which increases their manufacturing complexity and cost. Furthermore, these material systems could pose potential biotoxicity to the human body during usage and can also increase the environmental burden after usage. Hence, it still remains an unfilled research gap to achieve facile fabrication of fully passive and bio-friendly tactile sensing devices for detecting both static and dynamic stimulations.

**Fig. 1:**
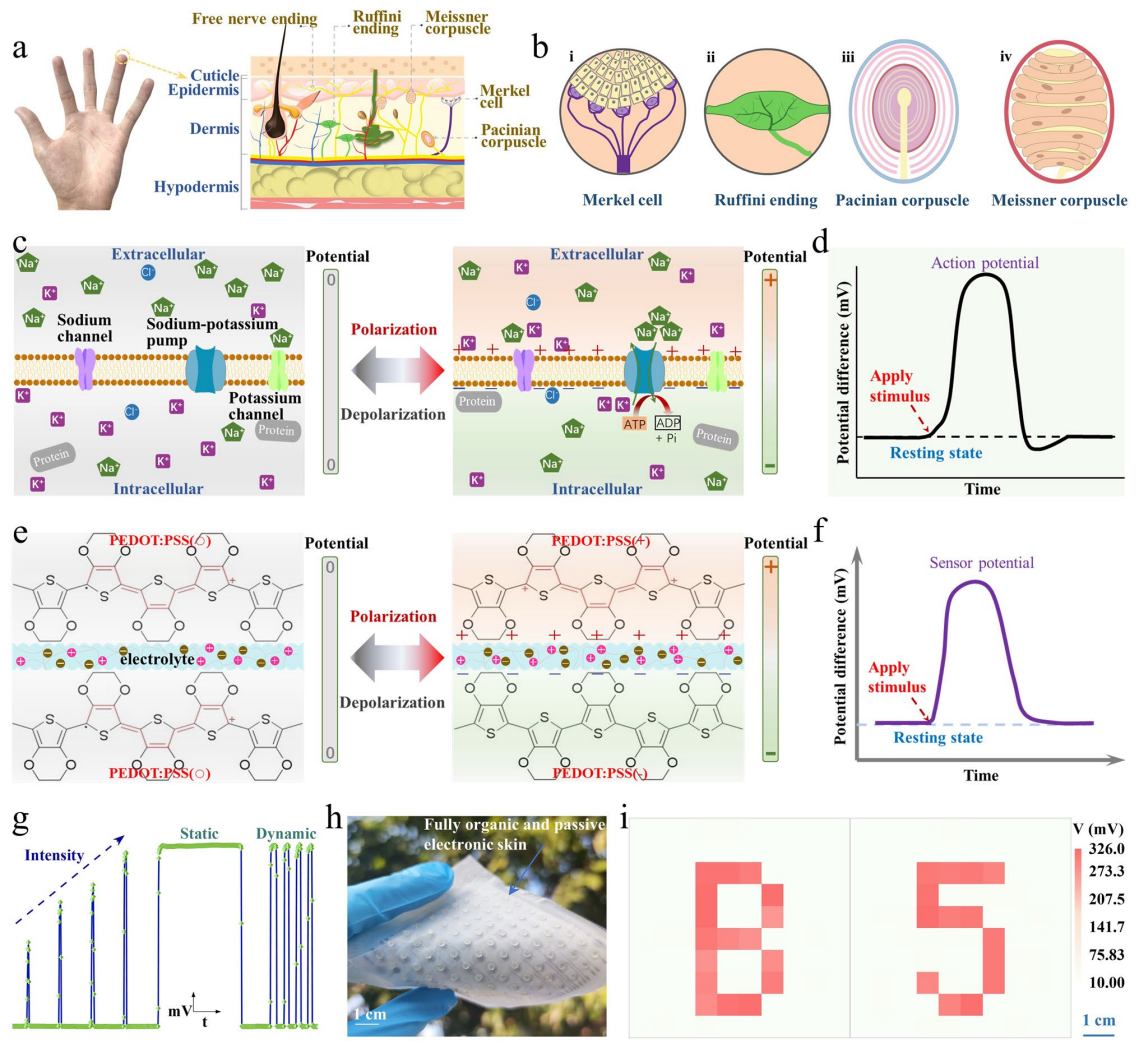
Bioinspired passive tactile sensation based on reversible polarization of conjugated polymers. **a, b** Illustrations showing the cutaneous mechanoreceptors in natural skin, including Merkel cells (i), Ruffini endings (ii), Pacinian corpuscles (iii), Meissner corpuscles (iv). **c** Schematics showing the polarization process of the sensory cells, with a potential difference established across the cell membrane. **d** Illustrative variation in potential difference across the cell membrane under tactile stimulations. **e** Schematics showing the polarization process of PEDOT:PSS into positively polarized PEDOT:PSS(+) and negatively polarized PEDOT:PSS(−), with a potential difference developed between the two electrodes. **f** Illustrative response behavior of the passive tactile sensors under tactile stimulations. **g** Recorded response signals of the passive tactile sensors when subjected to static and dynamic stimuli of different magnitudes. **h, i** Digital picture showing a piece of fully organic and passive tactile sensing electronic skin and its spatial tactile mapping capabilities. Scale bars, 1 cm

Natural skin is an energy-efficient sensory organ that is capable of perceiving diverse stimulations, including light touch, pressure, vibration, and pain [30]. An essential reason behind the energy efficiency of natural skin lies in its nearly passive sensing style. As shown in Fig. 1a, b, different sensory receptors can be found in natural skin, including Merkel cells, Ruffini endings, Pacinian corpuscles, Meissner corpuscles, etc. [31, 32]. These sensory cells can be transformed from a non-polarized state to a polarized state (Fig. 1c) by pumping specific ions across the cell membrane, giving rise to an electrical potential difference (i.e., membrane potential). Upon external stimulations, the ion channels in the cell membrane will be opened with ions flowing across the membrane, leading to a remarkable variation in the membrane potential (i.e., action potential, Fig. 1d) [33]. The membrane potential variations encoded with specific tactile information are finally transmitted to the brain cortex for tactile interpretation. Such passive tactile sensing process based on stimulus-triggered potential difference variations provides a highly effective but energy-efficient way to sense and perceive a wide spectrum of tactile stimulations.

Here, to mimic the natural tactile sensations, a new type of fully passive and bio-friendly tactile sensing devices is presented based on the unique reversible polarization chemistry of conjugated polymers. To emulate the polarization process of the sensory cells, an arbitrary conjugated polymer (such as poly (3, 4-ethylenedioxythiophene):poly (styrenesulfonate) (PEDOT:PSS), polyaniline, or polypyrrole) is polarized into two opposite states (i.e., positively polarized state and negatively polarized state), thus to develop an artificial potential difference (Fig. 1e). Then, solid ionic electrolytes with surficial microstructures are employed to imitate the ion channels, which could encode external touch stimulations into potential difference variations (i.e., sensor potential, Fig. 1f) measured between the two oppositely polarized conjugated polymers. This bioinspired tactile sensing methodology makes it possible to construct fully organic and bio-friendly touch sensing devices through greatly simplified process and minimized fabrication cost. The resultant tactile sensors exhibit high sensitivity (≈ 773 mV N^−1^), fast response and recovery rate (≈ 40 and ≈ 20 ms), ultralow power consumption (nW), desirable cyclic stability (over 5000 cycles), as well as good biocompatibility and degradability. More importantly, both static and dynamic tactile stimuli could be resolved with the proposed passive tactile sensors. As proof-of-concept demonstrations, single point tactile perception (including object surface texture perception and material property perception) as well as two-dimensional tactile recognitions (object shape or profile perception) are well demonstrated with the assistance of self-defined machine learning algorithms. This novel tactile sensing concept based on the reversible polarization chemistry of conjugated polymers opens up a new path toward endowing dexterous tactile sensing capabilities to smart prosthesis and intelligent robots.

## Experimental Section

### Materials

PEDOT:PSS was purchased from Agfa-Gevaert N.V. (Belgium). Conductive carbon ink (CH-8(MOD2)) was purchased from Jujo Chemical Co., Ltd. (Japan). 3-Glycidoxypropyl trimethoxysilane (GOPS) and pyrrole were purchased from Adamas-Beta (China). Sodium hydroxide, PVA (1788), Gly, NaCl, and *N,N*-dimethylformamide (DMF) were purchased from Chengdu Kelong Chemical Co., Ltd. (China). Phosphate buffer saline solution (PBS) was purchased from Beijing Labgic Technology Co., Ltd. (China). Aniline was purchased from Chengdu Jinshan Chemical Co., Ltd. (China). Hydrochloric acid was purchased from Sichuan Xilong Scientific Co., Ltd. (China). HEK-293 T cells were purchased from iCell Bioscience Inc. (China). CCK-8 was purchased from Invigentech (USA). Calcein-AM/PI double-stain kit was purchased from Bestbio (China). PLA films were purchased from Shenzhen Esun Industrial Co., Ltd. (China).

### Fabrication of the Passive Tactile Sensors with PEDOT:PSS

Fabrication of PEDOT:PSS(+) and PEDOT:PSS(−) electrodes: PLA films (40 µm thick) were used as the substrate for the sensors. Kapton tape films (50 µm in thickness) were laser-cut into defined patterns and attached to the PLA films as stencils. A layer of conductive carbon ink was applied to the stencil patterns, followed by drying at 60 °C for 60 min to cure the carbon ink. Then, the dried conductive carbon electrodes were exposed to the plasma (Sunjune VP-R, China) for 5 min to improve surficial hydrophilicity. Subsequently, 60 μL of aqueous PEDOT:PSS mixed solution, containing 92 wt% PEDOT:PSS, 5 wt% DMF, and 3 wt% GOPS, was applied to part A of the conductive carbon electrodes (as described in Fig. S1), followed by drying at 60 °C in the air oven for 20 min and then drying at 125 °C in the vacuum oven for 20 min to obtain PEDOT:PSS(○) electrodes. Finally, a source meter (Keithley 2601B, USA) was used to connect the two PEDOT:PSS(○) electrodes and apply a 1.5 V bias for 1 min in a 1 M NaCl solution, resulting in two oppositely polarized PEDOT:PSS, i.e., positively polarized PEDOT:PSS( +) and negatively polarized PEDOT:PSS(−). The two obtained PEDOT:PSS( +) and PEDOT:PSS(-) electrodes were used as the two electrodes of the passive tactile sensor after being left at room temperature for 1 h.

Preparation of micro-structured solid PVA/NaCl/Gly/H_2_O electrolytes: A 100 mM NaCl aqueous solution, a 15 wt% PVA aqueous solution, and a 12 wt% Gly aqueous solution were prepared. These solutions were mixed in different mass ratios (i.e., 1:10:1, 1:10:2, 1:10:4, and 1:10:8) to create 8%, 16%, 32%, and 64% PVA/NaCl/Gly aqueous solution. (X% means that the weight ratio of Gly to PVA is X%.) Next, 3 g of the above PVA/NaCl/Gly aqueous solutions were poured into a template with regular and periodic microstructures and then dried at 25 °C for a week in a well-ventilated area. After drying, the solid PVA/NaCl/Gly/H_2_O electrolyte films with regular surficial microstructures were peeled from the templates and cut into specific shapes and sizes. The micro-structured solid PVA/NaCl/Gly/H_2_O electrolyte (weight ratio of Gly to PVA is 32%) was used as the electrolyte of the passive tactile sensor.

Two PVA spacers (150 µm thick) were placed beside the sensing region of the electrodes, and the obtained micro-structured solid PVA/NaCl/Gly/H_2_O electrolyte films were put on the sensing region. Finally, the whole passive tactile sensor was encapsulated with PLA film (30 µm thick).

### Fabrication of Polyaniline and Polypyrrole Electrodes

Polyaniline and polypyrrole were synthesized using electrochemical deposition method. The electrochemical deposition was performed in a three-electrode-system setup on an electrochemical workstation (CHI660E, Shanghai Chenhua Company, China) with a Pt sheet electrode as the counter electrode, a saturated calomel electrode (SCE) as the reference electrode, and a carbon layer as the working electrode. For the fabrication of polyaniline, the precursor solution was prepared by mixing 0.2 M aniline and 1 M hydrochloric acid aqueous solutions. Electrochemical deposition was carried out using CV from − 0.2 to 0.9 V (vs. SCE) at a scan rate of 0.01 V S^−1^. For the fabrication of polypyrrole, the precursor solution was prepared by mixing an aqueous solution of 0.2 M pyrrole and 1 M hydrochloric acid. Electrochemical deposition was carried out using a CV from − 0.3 to 1.1 V (vs. SCE) at a scan rate of 0.03 V S^−1^. For the polarization of polyaniline or polypyrrole, two polyaniline or polypyrrole electrodes were subjected to a bias of 1.5 V for 1 min in a 1 M NaCl solution, resulting in two oppositely polarized polyaniline or polypyrrole electrodes.

### Electrochemical and Morphology Characterization of PEDOT:PSS

CV measurements were carried out on the electrochemical workstation (CHI660E, Shanghai Chenhua Company, China). The electrochemical performances were conducted using a three-electrode-system consisting of a counter electrode, a reference electrode, and a working electrode. A Pt sheet electrode was used as the counter electrode, a saturated calomel electrode (SCE) was used as the reference electrodes, and the investigated PEDOT:PSS(○) films were used as the working electrodes. The PBS solution was acted as the electrolyte. The CV curves were recorded between − 0.6 and + 0.6 V employing a scan rate of 0.03, 0.04, 0.05, and 0.06 V S^−1^. Surface morphology observation of the PEDOT:PSS(○), PEDOT:PSS(+), and PEDOT:PSS(−) was carried out with a scanning electron microscope (Apero S HiVac, FEI, USA).

### In Situ Characterization of the Polarization Processes of PEDOT:PSS

The polarization process of PEDOT:PSS is fully characterized by *in situ* monitoring its color change, Vis–NIR spectroscopy change, resistance change, and potential change. The measurement samples, setups, and methods are detailed in Table S1. In brief, two PEDOT:PSS(○) electrodes were subjected to a bias of 1.5 V for 1 min in a 1 M NaCl solution. During this process, the color changes, Vis–NIR spectroscopy changes, and potential difference variations (with respect to a PEDOT:PSS(○) electrode without bias) of the PEDOT:PSS(+) and PEDOT:PSS(−) are in situ measured and recorded continuously. After this process, the resistance changes of the PEDOT:PSS(+) and PEDOT:PSS(−) are recorded.

The repetitive and inverse polarization processes of PEDOT:PSS are illustrated in Fig. S2. The polarization process of PEDOT:PSS was conducted by applying a bias of 1.5 V to two PEDOT:PSS(○) electrodes in a 1 M NaCl solution for 1 min. The depolarization process was conducted by short-circuiting the two PEDOT:PSS(+) and PEDOT:PSS(−) electrodes for 1 min. The inverse polarization process of PEDOT:PSS was conducted by applying a bias of -1.5 V to the two PEDOT:PSS(○) electrodes in a 1 M NaCl solution for 1 min.

### Measurement of the Passive Tactile Sensors

The potential difference signals of our sensors were all collected using a source meter (Keithley 2601B). A custom-made mechanical testing setup with a stepping motor (KH-01, China), a motor controller, a sliding stage, and a digital force meter (Handpi-HLD, China) was used for the force measurements in the experiments. The mechanical frequency dynamic stimuli and stability tests were carried out on a swept-frequency signal generator (SA-SG030, ShiAo, China) integrated with a vibration exciter (SA-JZ002, ShiAo, China). All human subjects involved in the test on human bodies agreed to all tests and the picture in the manuscript with informed consent, and all tests were approved by the Scientific Ethical Committee of the School of Mechanical Engineering, Sichuan University.

### Cytotoxicity and Degradability Tests of the Passive Tactile Sensors

To assess the cytotoxicity of the passive tactile sensors, the sensors were immersed in a cell culture solution for 24 h. Subsequently, HEK-293 T cells were introduced into the cell culture solution. After a 24 h incubation, the cell culture solution was removed. Next, cell culture solution containing 10% CCK-8 was added to HEK-293 T cells. After 2 h of incubation, the cell viability was determined by measuring the absorbance at 450 nm using a microplate reader (SPARK 10 M, Tecan, Austria). For live/dead dyeing to evaluate the impact of the sensors on the death and viability of HEK-293 T cells, the cells incubated for 24 h in the cell culture solution were dyed using the Calcein-AM/PI double-stain kit for 15 min. Fluorescent images of the dyed viable and dead cells were captured using a confocal laser scanning microscope (Olympus FV1200, Japan). For degradability tests, the passive tactile sensors were immersed into a 0.5 M sodium hydroxide solution at 75 °C for 6 days. The photographs and weight measurements of the sensors were recorded every day.

### Machine Learning for Material Property Perception

A machine learning framework based on a 2D convolutional neural network (2D CNN) was employed to resolve, classify, and predict the material property curve patterns. The network consists of two convolutional layers, two max-pooling layers, and one fully connected layer. The convolutional layers use 5 × 5 convolutional kernel and zero-padding around the input feature maps. Following each convolution operation, the output of the convolutional layer is processed with the rectified linear unit (ReLU) activation function. Each max-pooling operation uses a 2 × 2 pooling kernel with a stride of 2. The output of the pooling layer is used as the input of the fully connected layer by unfolding. The number of output layer neurons corresponds to the different types of material property curves. The dataset is divided into a training dataset, which is used to train the 2D CNN machine learning framework, and a test dataset (with around a ratio of 5:5) used to predict and evaluate the network performance. Parameter optimization was carried out using stochastic gradient descent (SGD) with an initial learning rate of lr = 0.001 and a maximum training epoch of 8.

The recognition process of different materials and objects is elaborated in Note S1 and Figs. S3-S10. Firstly, the original raw data (i.e., potential difference vs time) were measured using a digital source meter (Keithley 2601B). The raw data were saved in an Excel format and were then transformed into two-dimensional images. The two-dimensional images were finally input into a 2D convolutional neural network for training and testing.

### Construction of Single-Electrode-Mode E-Skins

The fabrication of the single-electrode-mode e-skin is shown in Fig. S11. Conductive carbon inks were stencil printed on the PLA substrate. After drying the inks, a carbon layer was obtained. Then, an aqueous PEDOT:PSS mixed solution was applied to the carbon layer. After drying the solution and curing the PEDOT:PSS films, 10 × 10 small PEDOT:PSS(○) electrodes were used directly as sensing electrodes and 4 large PEDOT:PSS(○) electrodes were used as reference electrodes after being polarized to PEDOT:PSS(−) electrodes. A designed PVA spacer (50 µm in thickness) was placed above the electrodes, followed by placing micro-structured solid PVA/NaCl/Gly/H_2_O electrolyte (75 mm by 75 mm, weight ratio of Gly to PVA is 32%). Last a PLA film (30 µm in thickness) equipped with force collectors was put on the e-skin for encapsulation.

### Machine Learning for Object Shape Recognition

To evaluate the ability of e-skin to recognize shapes, a combined approach based on support vector machine (SVM) and principal component analysis (PCA) was employed to train and classify raw images. A total of 4 × 12 sets of data for four different shapes were collected and labeled. The samples were divided into a training and test set, which consisted of 20% of the data, while the remaining 80% formed the test set. The first step is to preprocess the images and extract feature information from them. Subsequently, PCA was utilized to reduce the image feature matrix to forty dimensions. Following this reduction, a multi-class classification model was trained using SVM on the training set data. This model transforms the multi-classification problem into a series of binary classification tasks, thereby constructing a classifier capable of handling multiple classes. Finally, we assessed the accuracy of this classifier based on the test set data.

The recognition process of different object shapes is elaborated in Note S2 and Figs. S12–S15. When an object was pressed on the electronic skin, the potential difference outputs measured between each of the sensing electrodes and the reference electrodes of the electronic skin were recorded one by one, and not measured simultaneously. Specifically, all reference electrodes were simultaneously connected to the reference electrode channel of the source meter (Keithley 2601B). Then, the working electrode channel of the source meter was successively connected to the sensing electrodes of the e-skin and the corresponding potential difference outputs from all of the sensing electrodes were manually recorded in a 10 × 10 table. Subsequently, the raw data were used to construct the two-dimensional color mappings. Finally, these two-dimensional color mappings were employed for training and testing.

## Results and Discussion

### Design Concept of Bioinspired Passive Tactile Sensation

The sensory cells in natural skin could transform from a non-polarized state to a polarized state (Fig. 1c), thus to establish a potential difference across the cell membrane. This polarization process provides the foundation for the passive sensing of external stimulations. At resting state, the potential difference across the cell membrane keeps constant. With a stimulation applied to the sensory cells, the cell membrane state would be altered, giving rise to a remarkable variation in the potential difference (Fig. 1d). In contrast to active sensing processes by continuously applying external energy to the devices, natural sensory cells feature a typical passive sensing process, i.e., stimulation event-triggered response outputs. This passive sensing style features much higher energy efficiency than that of active sensing methods.

To emulate the passive sensing process based on polarization of natural sensory cells, we take advantage of the reversible polarization capability of conjugated polymers to construct fully organic and passive tactile sensors. As a demonstration, PEDOT:PSS is selected and printed into two electrodes. The original non-polarized PEDOT:PSS is labeled as PEDOT:PSS(○). The two PEDOT:PSS(○) electrodes are then subjected to a bias of 1.5 V in an electrolyte solution, resulting in two oppositely polarized PEDOT:PSS, i.e., positively polarized PEDOT:PSS(+) and negatively polarized PEDOT:PSS(−). An artificial potential difference could be developed between the PEDOT:PSS(+) and PEDOT:PSS(−) electrodes (Fig. 1e). Then, a micro-structured solid ionic electrolyte is employed and set between the two oppositely polarized PEDOT:PSS electrodes. With this configuration, the electrode/electrolyte interfaces could be mechanically regulated, enables to encode external tactile stimuli into potential difference variations measured between the two polarized PEDOT:PSS electrodes (Fig. 1f).

The proposed passive tactile sensing devices are qualified for resolving both static and dynamic stimulations of different magnitudes (Fig. 1g). Fully organic and passive electronic skins could also be constructed for spatial tactile mapping (Fig. 1h, i). Notably, this touch sensing concept could be realized using different conjugated polymers (including PEDOT:PSS, polyaniline, and polypyrrole, Fig. S16), revealing the good adaptability and universality.

### Characterization of the Polarization Processes of PEDOT:PSS

**Fig. 2:**
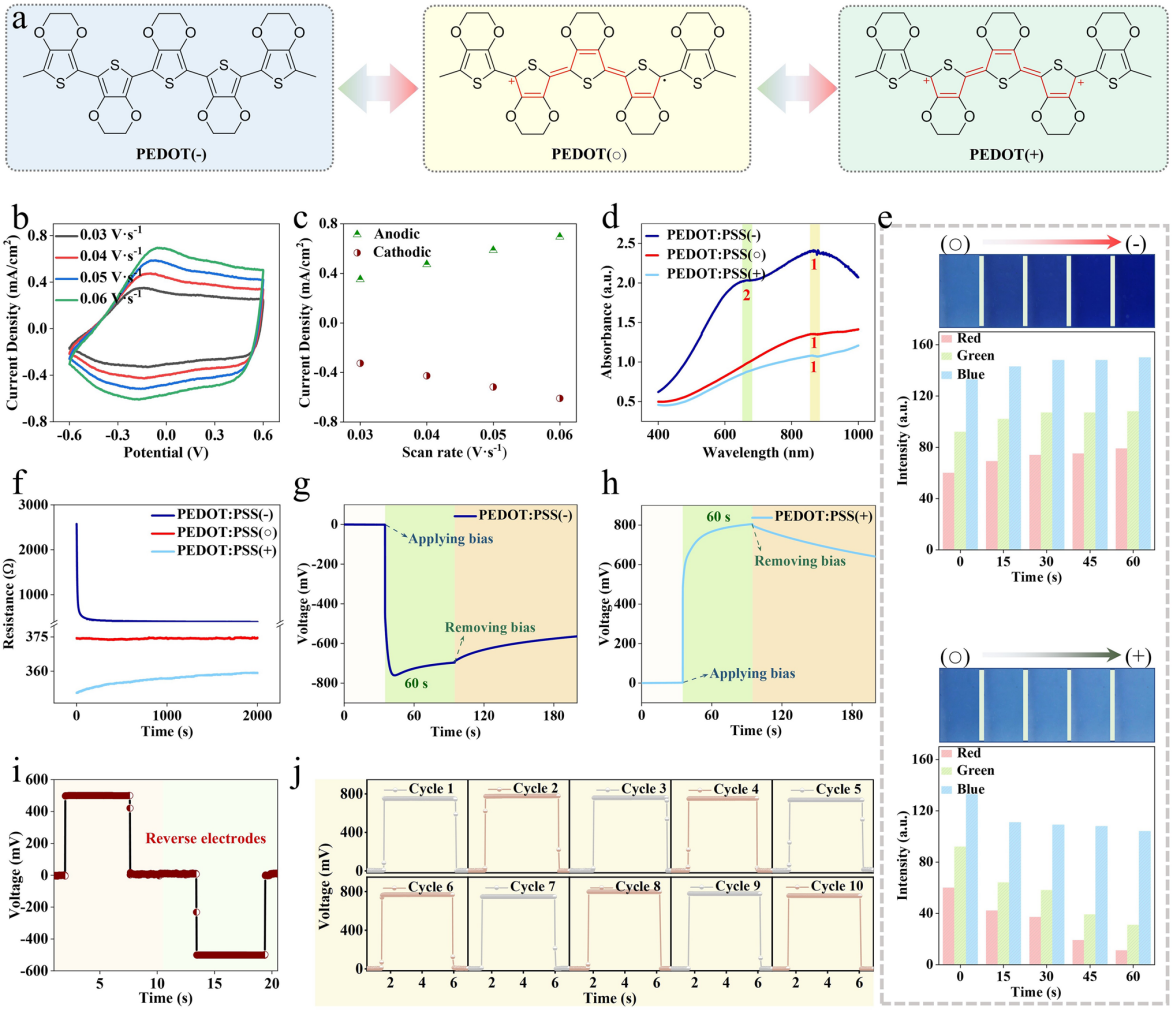
Characterization of the polarization processes of PEDOT:PSS. **a** Molecular structures of PEDOT in PEDOT:PSS(○), PEDOT:PSS(+), and PEDOT:PSS(−) states. **b** Cyclic voltammetry (CV) curves of PEDOT:PSS recorded at different scan rates. **c** Peak currents extracted from the CV curves at different scan rates. **d** Vis–NIR spectra of PEDOT:PSS(○), PEDOT:PSS(+), and PEDOT:PSS(−). **e** Digital pictures showing the color variations from PEDOT:PSS(○) to PEDOT:PSS( −) (top) and PEDOT:PSS(+) (bottom), respectively. **f** Resistance of PEDOT:PSS(○), PEDOT:PSS(+), and PEDOT:PSS(−), respectively. **g, h** Potential difference variations of PEDOT:PSS(−) (**g**), and PEDOT:PSS(+) (**h**) with respect to PEDOT:PSS(○) during the bias process, followed by removing the bias. **i** Potential difference measured between PEDOT:PSS(−) and PEDOT:PSS(+), followed by inverse polarization of these two electrodes. **j** Potential differences measured between PEDOT:PSS(−) and PEDOT:PSS(+) during repeated polarization, depolarization, and repolarization processes for 10 cycles

PEDOT:PSS is the most investigated conjugated polymer with mixed ion–electron conducting characteristics. The chemical structure of PEDOT:PSS is shown in Fig. S17, which consists of active PEDOT backbone and dopant PSS side chains. The doping states of PEDOT could be arbitrarily and continuously mediated (Fig. 2a). The neutral state PEDOT:PSS(○) could be transformed to less doped state PEDOT:PSS(−) by negative polarization, or to more doped state PEDOT:PSS(+) by positive polarization.

The reversible polarization process of PEDOT:PSS is characterized as shown in Fig. 2b-j. PEDOT:PSS has both electrical conductivity and electrochemical activity. Thus, the polarization process involves both capacitive behavior and Faradaic behavior [34–36]. Capacitive polarization processes store charges at an electric double layer and are characterized by a rectangle-shaped cyclic voltammetry (CV) curve. In contrast, Faradaic polarization process involves charge transfer across the interfaces and results in distinct redox peaks in the CV curve (as described in Fig. S18). In this study, both rectangle-shaped features and redox peaks in CV curves are observed (Fig. 2b), revealing both capacitive and Faradaic polarizations of PEDOT:PSS. Higher peak currents are measured under higher scan rates (Fig. 2c), which agrees with the capacitive as well as Faradaic polarization behaviors.

The existence of different polarization states of PEDOT:PSS is verified via Vis–NIR spectroscopy. As shown in Fig. 2d, the spectrum of original PEDOT:PSS(○) displays a peak at 860 nm, which can be attributed to the polaron absorption [37, 38]. After positively polarized, the spectrum of PEDOT:PSS(+) exhibits a lower peak at 860 nm. This is due to that part of the polarons are oxidized to bipolarons during the positive polarization process. In contrast, after negative polarization, the spectrum of PEDOT:PSS(−) exhibits a much higher peak at 860 nm due to the reduction of partial bipolarons into polarons. Additionally, a new peak at about 660 nm is observed, which arises from the further reduction of polarons [36]. These results reflect the variation of PEDOT:PSS during to the polarization process.

Moreover, the polarization process of PEDOT:PSS is in situ characterized by continuously monitoring its color change, resistance change, and potential change. Figures 2e and S19 show the color change of PEDOT:PSS from blue to light blue during positive polarization. Conversely, the color of PEDOT:PSS changes from blue to dark blue during negative polarization. These results agree well with the Vis–NIR spectroscopy observation. The continuous resistance change of PEDOT:PSS after the reversible polarization process is presented in Fig. 2f. The resistance shows dramatic decrease during positive polarization because more charge carriers are generated. On the contrary, during negative polarization, the resistance exhibits a slight increase. The potential difference variations of PEDOT:PSS( +)-PEDOT:PSS(○) as well as PEDOT:PSS(−)-PEDOT:PSS(○) are also recorded during the polarization process. As shown in Fig. 2g, h, positive polarization elevates the potential of PEDOT:PSS, while negative polarization leads to the decline in the potential of PEDOT:PSS. The surface morphology of PEDOT:PSS does not show obvious difference (Fig. S20), indicating that the polarization process only affects the molecular structure rather than the morphology of PEDOT:PSS.

Via the polarization process, potential difference outputs with different values could be created between PEDOT:PSS(○), PEDOT:PSS(+), and PEDOT:PSS(−) (Fig. S21). Notably, the polarization of PEDOT:PSS is invertible and reversible. As shown in Fig. 2i, PEDOT:PSS(+) and PEDOT:PSS(−) could be inverted when subjected to an inverse polarization process, with opposite potential difference measured. Moreover, the polarization and depolarization process could be repeated with good reproducibility (Fig. 2j). It is worth pointing out that the PEDOT:PSS(−) and PEDOT:PSS( +) states are not highly stable and are prone to go back to the PEDOT:PSS(○) state when exposed to air (Fig. S22), especially at high temperature. After a long period of time, a repolarization process is needed to keep a desirable intensity of the sensor signals.

### Performance Evaluations of the Passive Tactile Sensors

**Fig. 3:**
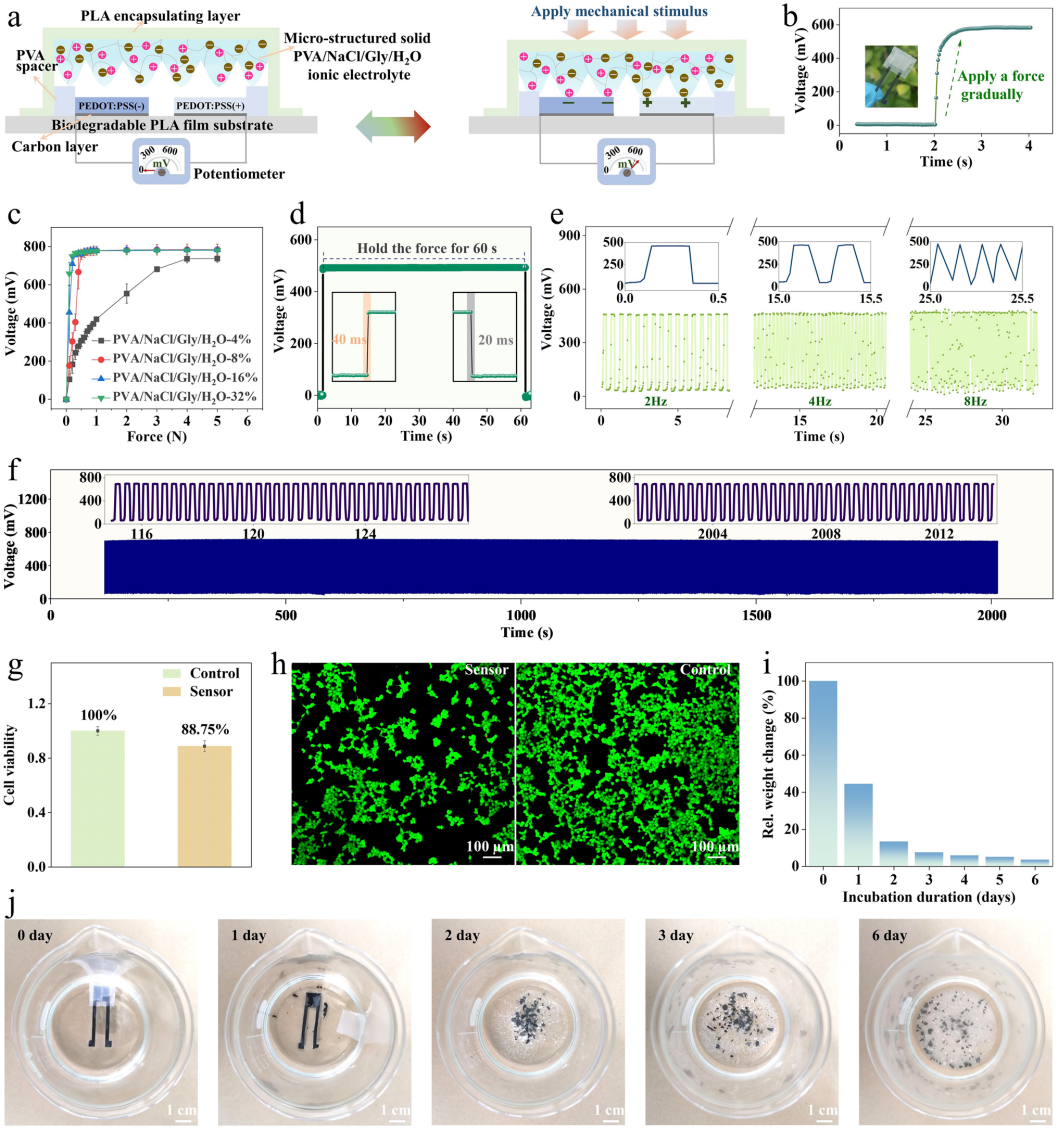
Configuration and performance of the passive tactile sensors. **a** Schematics illustrating the cross-sectional configurations of the passive tactile sensors before and after applying a force on the devices. **b** Potential difference output of a sensor while gradually applying a force on the device. **c** Response behaviors of the sensors with micro-structured solid PVA/NaCl/Gly/H_2_O electrolytes of different Gly contents. Here, PVA/NaCl/Gly/H_2_O-X% means that the weight ratio of Gly to PVA is X%. **d** Response and recovery behaviors of the tactile sensors. And continuously recorded signal output of the sensors when holding a force for 60 s, verifying the ability of the sensors to detect static stimuli. **e** Potential difference variations of the sensors when applying dynamic forces of difference frequencies (i.e., 2, 4, and 8 Hz, respectively), indicating the capability of the sensors to monitor dynamic stimuli. **f** Reliability test of the sensors by loading and unloading a force on the devices for 5000 cycles. **g** Cell viability test (with HEK-293T cells, CCK-8 assay) of the tactile sensors after immersion in cell culture solution for 24 h. **h** Fluorescent images showing the dyed viable (green) and dead (red) HEK-293T cells (with Calcein-AM/PI double-stain assay) acquired from the experimental sample (i.e., with sensors) and control sample (i.e., without sensors). Scale bars, 100 µm. **i** Relative mass change of the tactile sensors after immersed in sodium hydroxide solution (0.5 M) for 6 days. **j** Photographs showing the degradation process of the passive tactile sensors. Scale bars, 1 cm

The reversible polarization of PEDOT:PSS allows to create a potential difference, which could be further mechanically regulated with a micro-structured ionic electrolyte. Via this mechanism, fully organic and passive touch sensing devices could be constructed. The configuration of the passive tactile sensors is shown in Figs. 3a and S23. A micro-structured ionic electrolyte composed of polyvinyl alcohol (PVA), glycerol (Gly), sodium chloride (NaCl), and water (H_2_O) is set between the PEDOT:PSS(+) and PEDOT:PSS(−) electrodes. Tactile stimulations could regulate the electrode/electrolyte interfaces, resulting in variations in the potential difference outputs measured between the two electrodes.

As shown in Fig. 3b, the passive tactile sensors show a continuous and smooth upswing in the signal output when applying a mechanical force gradually. The response behaviors of the sensors could be modulated by tuning the Gly content in the PVA/NaCl/Gly/H_2_O ionic electrolytes (Fig. 3c). Gly acts as a humectant and can tune the water content and electrical impedance of the ionic electrolytes. Additionally, Gly also serves as a plasticizer that can increase the softness of the ionic electrolytes. Sensors with electrolyte of higher Gly content exhibit higher sensitivity and lower detection limit, while sensors with electrolyte of lower Gly content show a broader detection range. For instance, in the force range of 0–1 N, the sensors with PVA/NaCl/Gly/H_2_O-32% electrolyte shows much higher sensitivity (≈773 mV N^−1^) than that of sensors with PVA/NaCl/Gly/H_2_O-4% solid electrolyte (≈420 mV N^−1^). The response and recovery speeds of the sensors are measured to be ≈ 40 and ≈ 20 ms, respectively (Fig. 3d). Notably, the power consumption of the passive sensors is ultralow and was recorded to be < 1 nW (Fig. S24).

The passive tactile sensors also have good capability to resolve both static and dynamic simulations. As shown in Fig. S25, when forces of different intensities are applied on the sensors, the signal outputs display distinct responses, showing the capability of the sensors to detect force variations continuously. Notably, unlike traditional resistive, capacitive, and transistor-based sensors, the signal outputs of these sensors are totally self-generated without external power supply. The self-powered capability of the sensors is demonstrated in Fig. S26. When compared to triboelectric and piezoelectric sensing devices that selectively respond to dynamic stimuli, these sensors show superiority in continuously monitoring both static and slowly varying stimuli. As shown in Fig. 3d, when a static force is applied on the sensors and maintained for 60 s, the recorded signal output remains nearly constant during this period. The sensors are also capable of monitoring dynamic stimuli up to 8 Hz, as demonstrated in Fig. 3e. Additionally, the sensors exhibit desirable durability in a cyclic test over 5000 cycles (Fig. 3f). All of the results demonstrate the good capabilities of the passive sensors for resolving complex tactile stimulations. It is worth mentioning that the sensor performance is sensitive to bending deformation, but the bending deformation would not damage the sensor structures (Fig. S27).

The proposed passive tactile sensors are composed of fully organic and bio-friendly materials (e.g., PEDOT:PSS, PVA, polylactic acid (PLA), Gly, and carbon), exhibiting superior biocompatibility and degradability. This is of great significance for the safe application and harmless disposal of the sensors.

The biocompatibility of the sensors is assessed through a cytotoxicity test using the Cell Counting Kit-8 (CCK-8) assay. As shown in Fig. 3g, the cell viability of HEK-293 T cells calculated from the culture solution containing the sensors is 88.75% with respect to the control group, meeting the recommended 70% cell viability threshold by USP (ISO 10993-5) [39]. This result suggests that the sensors are nearly non-toxic and biocompatible with biological cells. Additionally, the cytotoxicity of the sensors is further verified using Calcein-AM/PI double-stain assay (Fig. 3h). The fluorescent images show that the sensors have good biological safety.

The passive tactile sensors also feature good degradability and harmlessness to the environment. The mass loss kinetics of the sensors in alkaline aqueous environment is investigated. As shown in Fig. 3i, j, by immersing the sensor into 0.5 M sodium hydroxide solution for 6 days, the vast majority (≈ 96 wt%) of sensor composition (e.g., PVA and PLA) is dissolved or degraded [40, 41]. A small amount of PEDOT:PSS and carbon (≈ 4 wt%) are left during the short degradability process, which, nevertheless, could be decomposed and degraded in nature in the long term [42, 43].

### Single Point Tactile Sensation and Perception

The mechanoreceptors in natural skin have both slow adapting and fast adapting capability, able to acquire rich tactile information including surface texture and material properties. Here, we use the developed passive tactile sensors with both static and dynamic sensing capabilities to imitate the functions of natural skin.

**Fig. 4:**
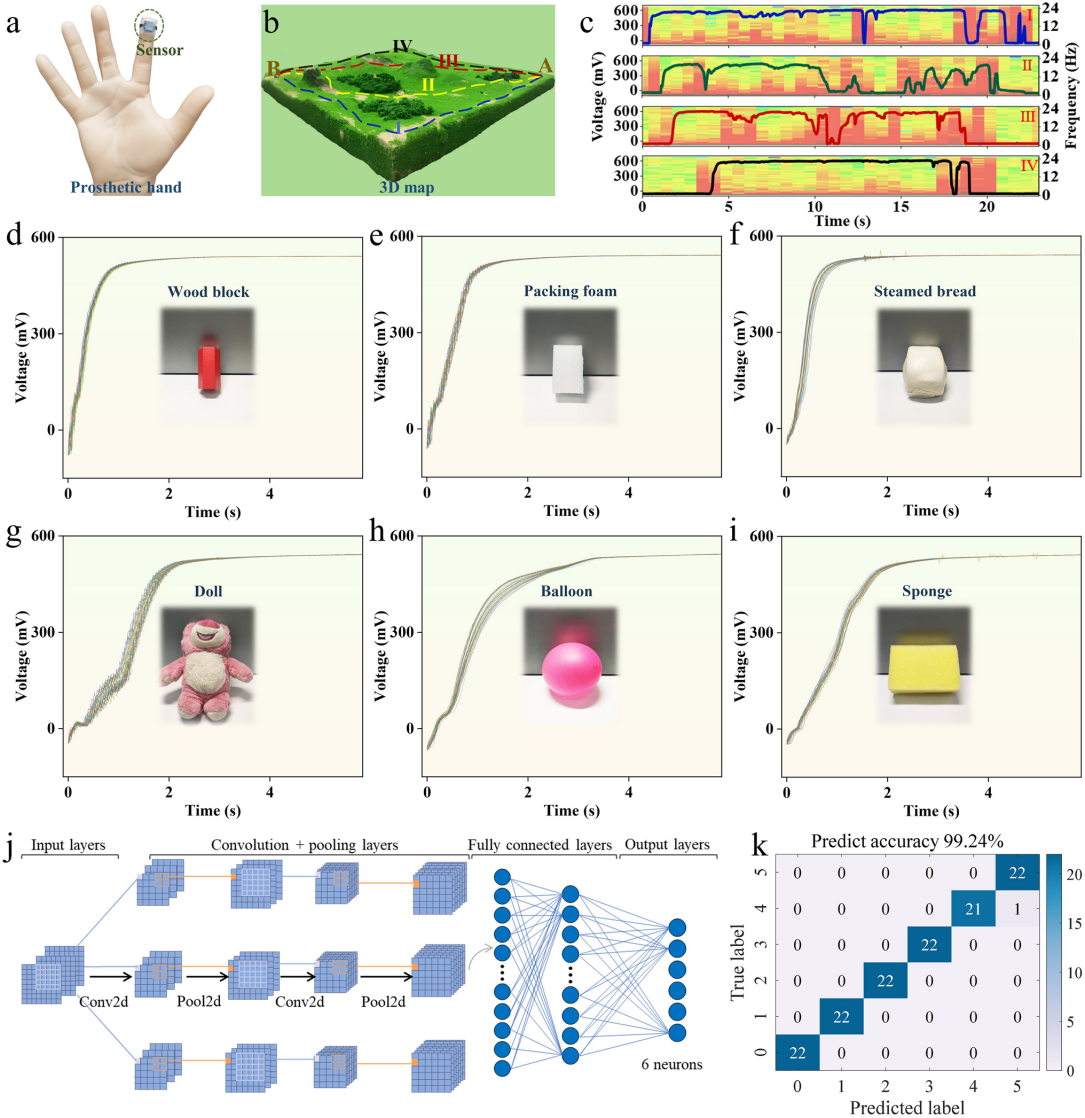
Surface texture and material property recognition with the passive tactile sensors. **a** Photograph showing a tactile sensor attached to the fingertip of a prosthetic hand. **b** Photograph illustrating the four sliding paths (i.e., I, II, III, and IV) on a 3D map model from point A to point B. **c** The recorded potential difference variations and the extracted STFT spectra when the fingertip slides along path I, II, III, and IV, respectively. **d**–**i** The recorded potential difference signals when the fingertip equipped with the sensor touches different objects, including wood block (**d**), packing foam (**e**), steamed bread (**f**), doll (**g**), balloon (**h**), and sponge (**i**) for 30 times. **j** Schematics illustrating the machine learning framework based on a 2D convolutional neural network (2D CNN). **k** Confusion matrix showing the prediction results of objects with different material properties. 0-wood block, 1-packing foam, 2-steamed bread, 3-doll, 4-balloon, 5-sponge

As proof-of-concept demonstrations, a passive tactile sensor is integrated onto the fingertip of a prosthetic hand to detect the texture of different objects (Fig. 4a). The fingertip equipped with the tactile sensors slides on a 3D map with complex surface texture from point A to point B through different paths (i.e., I, II, III, and IV, Fig. 4b). As presented in Fig. 4c, sliding along different paths gives rise to distinct sensor signal variations due to the different surface texture on different paths. Short-time Fourier transform (STFT) spectra extracted from the response signals show more clear difference of the surface texture. In the rugged area, the sensor signals exhibit more dramatic variations, while flat areas give rise to stable signal outputs. Besides, a keyboard is used to verify the surface texture sensing capability of the sensors (Fig. S28).

In addition to surface texture sensation, it is an essential ability for nature skin to perceive and distinguish the material properties of different objects. As a demonstration, the passive sensors are used to perceive the material properties of a diversity of daily objects, including wood block, packing foam, steamed bread, doll, balloon, sponge, etc. The experimental setup is shown in Fig. S29. As shown in Fig. 4d-i, when different objects are touched by the sensors in the same manner, the response signals from the sensors show a similar general trend, but have different subtle characteristics. These distinct signal characteristics could be attributed to the significant difference in the material properties, including softness, compressibility, constitution, porosity, and so on. These subtle characteristics are complicated and are difficult to be recognized by the naked eyes. Hence, a machine learning framework based on a 2D convolutional neural network (2D CNN) is employed to resolve and classify these signal characteristics, thus to achieve better recognition of different materials and objects.

As shown in Fig. 4j, the machine learning network consists of two convolutional layers, two max-pooling layers, and one fully connected layer. The dataset is divided into a training dataset and a test dataset. The training dataset is used to train the 2D CNN machine learning framework, and the test dataset (with around a ratio of 5:5) used to predict and evaluate the network performance. Figure 4k shows the confusion matrix of the machine learning results for prediction of objects with different material properties. The result reveals that the 2D CNN model has a high prediction accuracy of 99.24%, verifying the good reliability of the machine learning framework as well as the passive tactile sensors.

The passive tactile sensors are also qualified to monitor wrist pulse (Fig. S30). For example, a prosthetic hand equipped with the passive sensors could be used to detect the wrist pulse and the heart rate variations, indicating good potential for healthcare applications.

### Two-Dimensional Tactile Sensation and Recognition

**Fig. 5:**
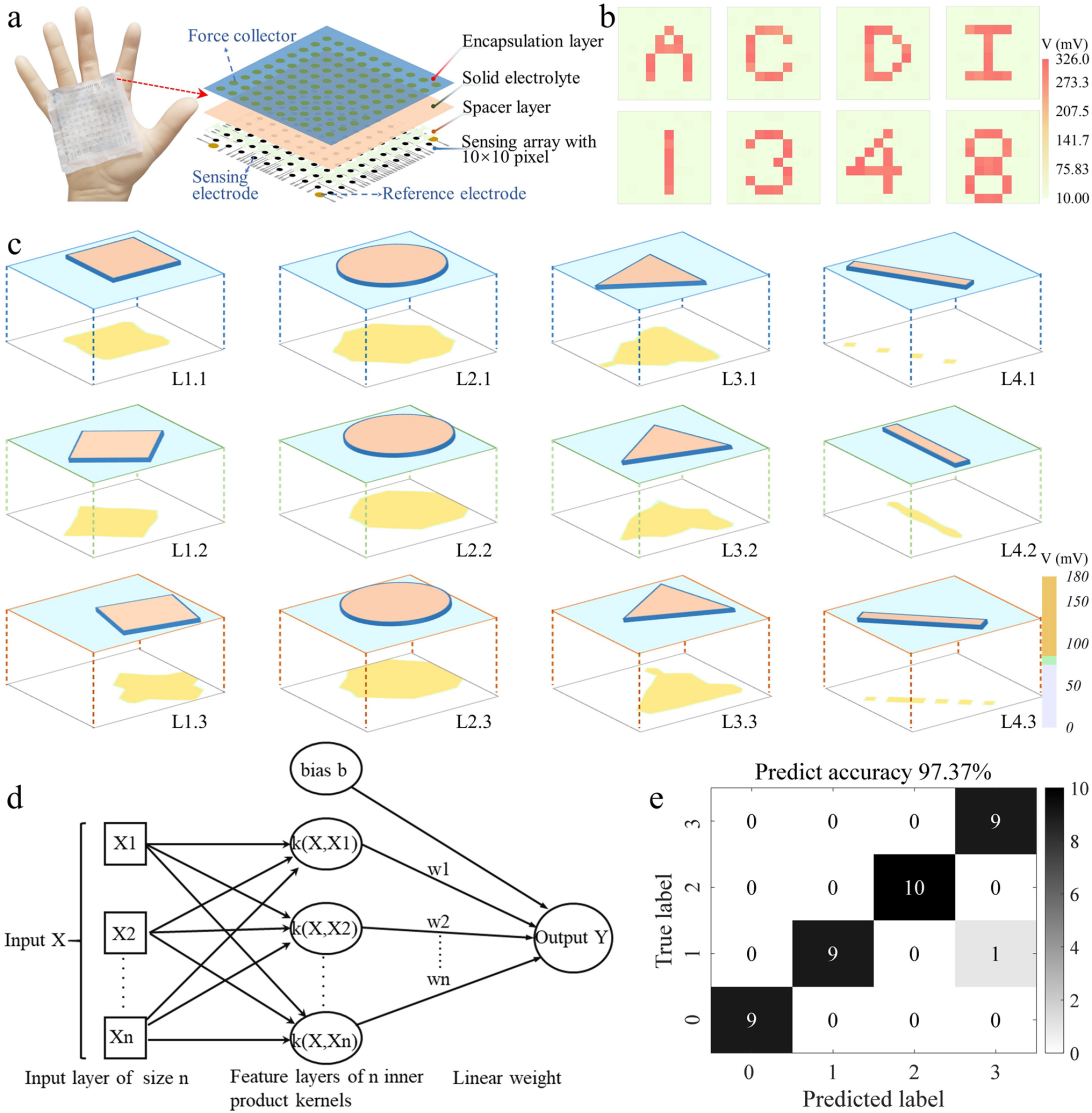
Passive and single-electrode-mode e-skin for high accuracy shape recognition. **a** Schematic components and layout of the single-electrode-mode e-skin. **b** Spatial mappings in the potential difference outputs of each sensing pixel with respect to the reference electrodes when 3D printed molds with different shapes are pressed onto the e-skin. **c** Schematic diagrams and reconstructed color mapping images when objects with different shapes (i.e., cube, cylinder, triangular prism, and rod) are placed to the e-skin at different positions and in different angles. **d** Schematic illustrating the machine learning framework of radial basis function (RBF)-based support vector machine (SVM). **e** Confusion matrix showing the shape recognition results. 0-cube, 1-cylinder, 2-rod, 3-triangular prism

With large number of mechanoreceptors, natural skin has the capability to perceive the geometrical shape and profile of different objects. Here, a kind of fully organic, passive, and single-electrode-mode electronic skin (e-skin) is developed to imitate the two-dimensional tactile mapping ability of natural skin. The structure layout of the passive e-skin is illustrated in Fig. 5a. The detailed fabrication process is elaborated in the experimental section and Fig. S11. Briefly, conductive carbon inks are stencil printed on the flexible PLA substrate, forming the backing electrodes and connecting wires. Then, PEDOT:PSS is applied to the functional areas of the sensing electrodes and reference electrodes. The PEDOT:PSS of the reference electrodes is then negatively polarized to PEDOT:PSS(−), thus to develop potential differences between the sensing electrodes and the reference electrodes. A PVA spacer layer (50 µm) is set between the electrodes layer and the micro-structured solid electrolyte. Finally, the entire e-skin is encapsulated with a PLA layer. An array of force collectors is set upon the outside of encapsulation layer to enhance the sensitivity of the passive e-skin.

This passive and single-electrode-mode e-skin features two advantages compared to conventional e-skins. Firstly, the passive e-skin includes 10 × 10 sensing electrodes and four reference electrodes. So, there are totally 100 sensing units and only 104 electrodes, which greatly alleviates the complex wiring issues and fabrication complexity. Secondly, the operation of the passive e-skin is based on measuring the electrical potential differences between all of the sensing electrodes with respect to the reference electrodes. Notably, there is nearly no current passing through the measuring circuit for the potential difference measurement. So, the interference and cross-talk between different sensing pixels could be significantly minimized (Fig. S31).

To evaluate the profile mapping capability of the passive e-skin, 3D printed plastic molds with different letter shapes (i.e., A–J) and number shapes (i.e., 0–9) are pressed to the e-skin. The potential difference outputs of each sensing pixels with respect to the reference electrodes are measured, and their two-dimensional color mappings are constructed. As presented in Figs. 5b and S32, the reconstructed color mapping of the output signals agrees well with the practical shapes of the letters and numbers, demonstrating the capability of the e-skin for resolving two-dimensional tactile profiles.

To further demonstrate the shape recognition capability of the passive e-skin, four objects of different shape (e.g., cube, cylinder, triangular prism, and rod, labeled as L1–L4) were selected. The four objects are arbitrarily pressed to the e-skin at different positions and in different angles. The profile mappings of all the above-mentioned situations are presented in Figs. 5c and S33, with more than 4 × 12 sets of color mappings reconstructed. It is noted that, even with the same object, the reconstructed color mappings exhibit substantial differences when the object is placed on the e-skin in different positions and angles. Based on the prominent variations in the reconstructed mappings, an image-based machine learning framework is developed to recognize and classify these mappings. The image-based machine learning framework based on support vector machine (SVM) and principal component analysis (PCA) is shown in Fig. S34.

For all of the acquired mappings, 20% of them are used as the training set, and the other 80% act as the test set. The first step involves preprocessing the images and extracting the feature information from them. Subsequently, PCA is used to reduce the image feature matrix to forty dimensions. After this dimension reduction, a multi-class classification model is trained using SVM on the training set data (Fig. 5d). Finally, the accuracy of this classifier is assessed based on the test set data. Figure 5e shows the shape recognition result via the PCA-SVM model. A high accuracy of 97.37% is achieved, verifying the good reliability of the machine learning framework and the desirable performance of the single-electrode-mode e-skin.

## Conclusions

In summary, we have demonstrated a new paradigm of fully organic and bioinspired passive touch sensors based on reversible polarization of single conjugated polymers (including PEDOT:PSS, polyaniline, and polypyrrole). The reversible polarization processes and polarization mechanisms of PEDOT:PSS are fully investigated and discussed. Taking advantage of the reversible polarization of PEDOT:PSS, potential difference variations could be created and encoded with tactile stimulations, resulting in a new bioinspired passive tactile sensing mechanism. The resultant passive tactile sensors feature ultralow energy consumption (nW), high sensitivity (773 mV N^−1^), rapid response/recovery times (≈ 40 and ≈ 20 ms), good reproducibility (over 5000 cycles), and, most importantly, unique ability to monitor both static and dynamic stimulations. As promising applications, single point tactile sensations including surface texture perception and material property perception are well demonstrated. Furthermore, a type of fully organic, single-electrode-mode, and passive e-skin is developed and exhibits high accuracy (97.37%) for two-dimensional shape recognitions with the assistance of machine learning algorithms. This presented work provides both new sensing mechanisms as well as new technological approaches for mimicking the natural tactile sensing functionalities and could advance the development of intelligent prosthesis and robots.

## Supplementary Information

Below is the link to the electronic supplementary material.

Supplementary file1 (DOCX 7848 kb)
