# Supplementary material for: Bioinspired Passive Tactile Sensors Enabled by Reversible Polarization of Conjugated Polymers
Source: Nanomicro Lett. 2024 Sep 27;17:16. doi: 10.1007/s40820-024-01532-z (PMC11427634; doi:10.1007/s40820-024-01532-z)
Supplement: Supplementary file 1 — Supplementary file1 (DOCX 7848 kb) [file 40820_2024_1532_MOESM1_ESM.docx]

Supporting Information for

**Bioinspired Passive Tactile Sensors Enabled by Reversible Polarization of Conjugated Polymers**

Feng He^1^, Sitong Chen^1^, Ruili Zhou^1^, Hanyu Diao^2^, Yangyang Han^3,^*, and Xiaodong Wu^1,^*

^1^School of Mechanical Engineering, Sichuan University, Chengdu 610065, P. R. China

^2^School of Software Engineering, Chongqing University of Posts and Telecommunications, Chongqing 400065, P. R. China

^3^State Key Laboratory of Polymer Materials Engineering, Sichuan University, Chengdu 610065, P. R. China

*Corresponding authors. E-mail: [hyyscu@hotmail.com](mailto:hyyscu@hotmail.com) (Yangyang Han); [xiaodong_wu@scu.edu.cn](mailto:xiaodong_wu@scu.edu.cn) (Xiaodong Wu)

**Supplementary Figures**


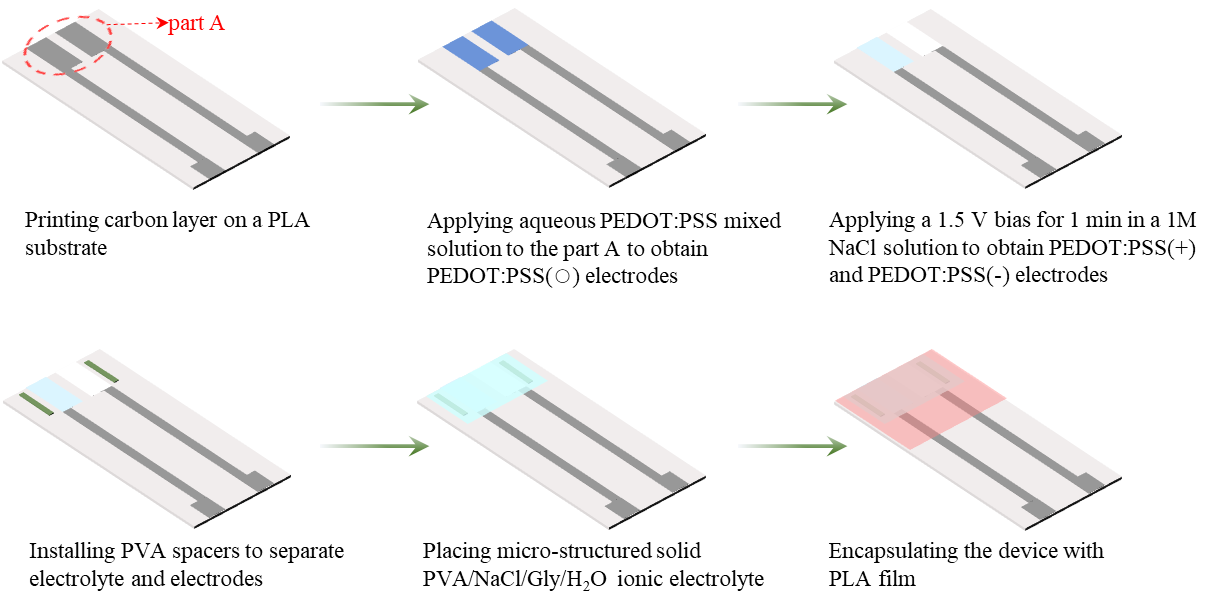


**Fig. S1** Fabrication process of the passive tactile sensors

**Table S1** *In-situ* characterizations of the polarization processes of PEDOT:PSS with different methodologies, including spectroscopy change measurement, color change observation, resistance change recording, and potential change measurement

| **Characterization methods** | **Diagrams of the samples and setups** | **Experimental methods** |
| --- | --- | --- |
| In-situ color change observation | 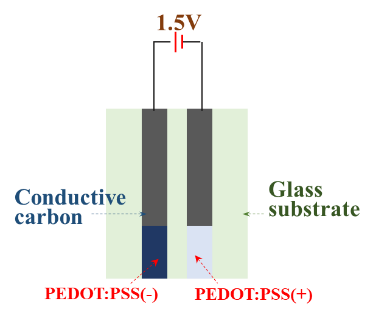 | Two PEDOT:PSS(○) electrodes were subjected to a bias of 1.5 V for 1 min in a 1 M NaCl solution. During this process, the color changes from PEDOT:PSS(○) to PEDOT:PSS(+) and PEDOT:PSS(-) were *in-situ* observed with an optical microscope (SN-300, China). |
| In-situ Vis-NIR spectroscopy observation | 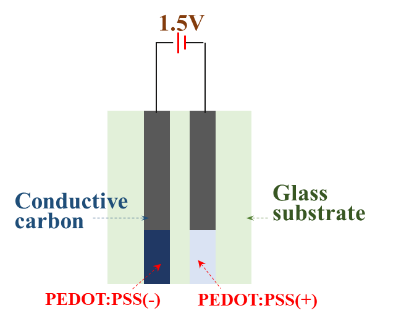 | Two PEDOT:PSS(○) electrodes were subjected to a bias of 1.5 V for 1 min in a 1 M NaCl solution. During this process, the Vis-NIR spectroscopy changes from PEDOT:PSS(○) to PEDOT:PSS(+) and PEDOT:PSS(-) were *in-situ* characterized with a Vis-NIR photometer (UV-3600, Shimadzu, Japan). |
| In-situ resistance recording | 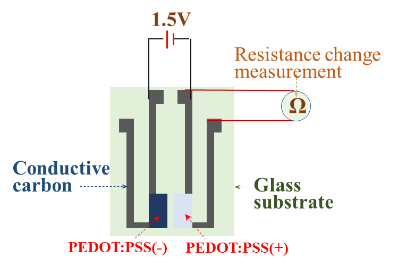 | After the two PEDOT:PSS(○) electrodes were biased (at 1.5 V for 1 min in 1M NaCl solution), the resistance changes of the PEDOT:PSS(+) and PEDOT:PSS(-) were recorded with a source meter (Keithley 2601B, USA) through another two connecting wires. |
| In-situ  potential difference recording | 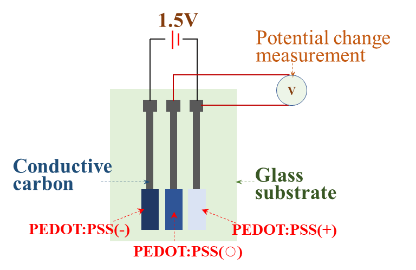 | One PEDOT:PSS(○) electrode was used as a reference electrode. Another two PEDOT:PSS(○) electrodes were subjected to a bias of 1.5 V for 1 min in a 1 M NaCl solution. During this process, the potential difference variations of PEDOT:PSS(+) and PEDOT:PSS(-) with respect to the PEDOT:PSS(○) reference electrode were *in-situ* measured with a source meter (Keithley 2601B, USA). |


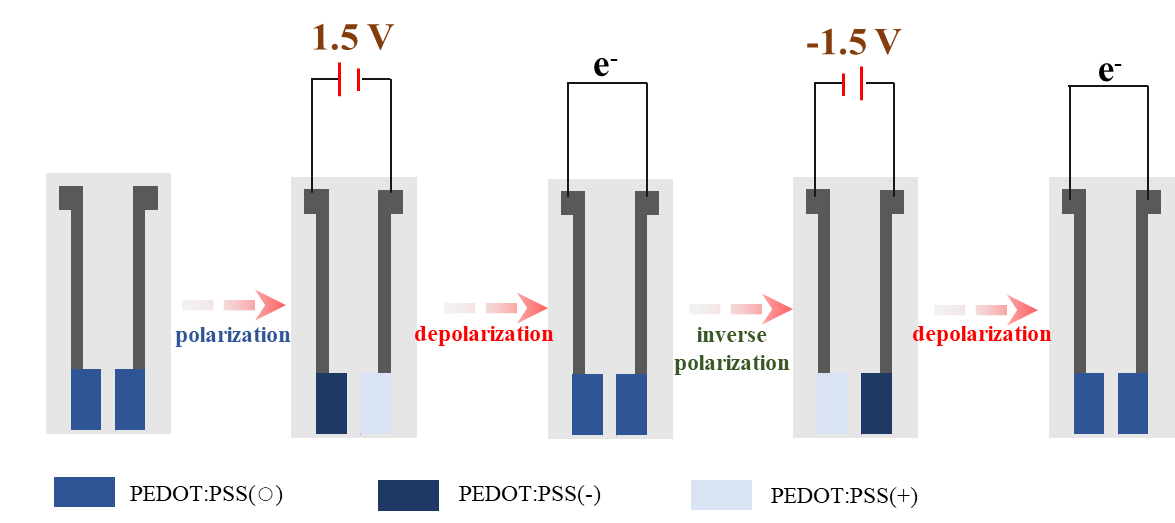


**Fig. S2** Schematic illustrations showing the polarization, depolarization and inverse polarization processes of PEDOT:PSS electrode. The polarization process of PEDOT:PSS was conducted by applying a bias of 1.5 V to two PEDOT:PSS(○) electrodes in a 1 M NaCl solution for 1 min. The depolarization process was conducted by short-circuiting the two PEDOT:PSS(+) and PEDOT:PSS(-) electrodes for 1 min. The inverse polarization process of PEDOT:PSS was conducted by applying a bias of -1.5 V to the two PEDOT:PSS(○) electrodes in a 1 M NaCl solution for 1 min

**Note S1 Data collection and processing methods for single point tactile sensation**

**
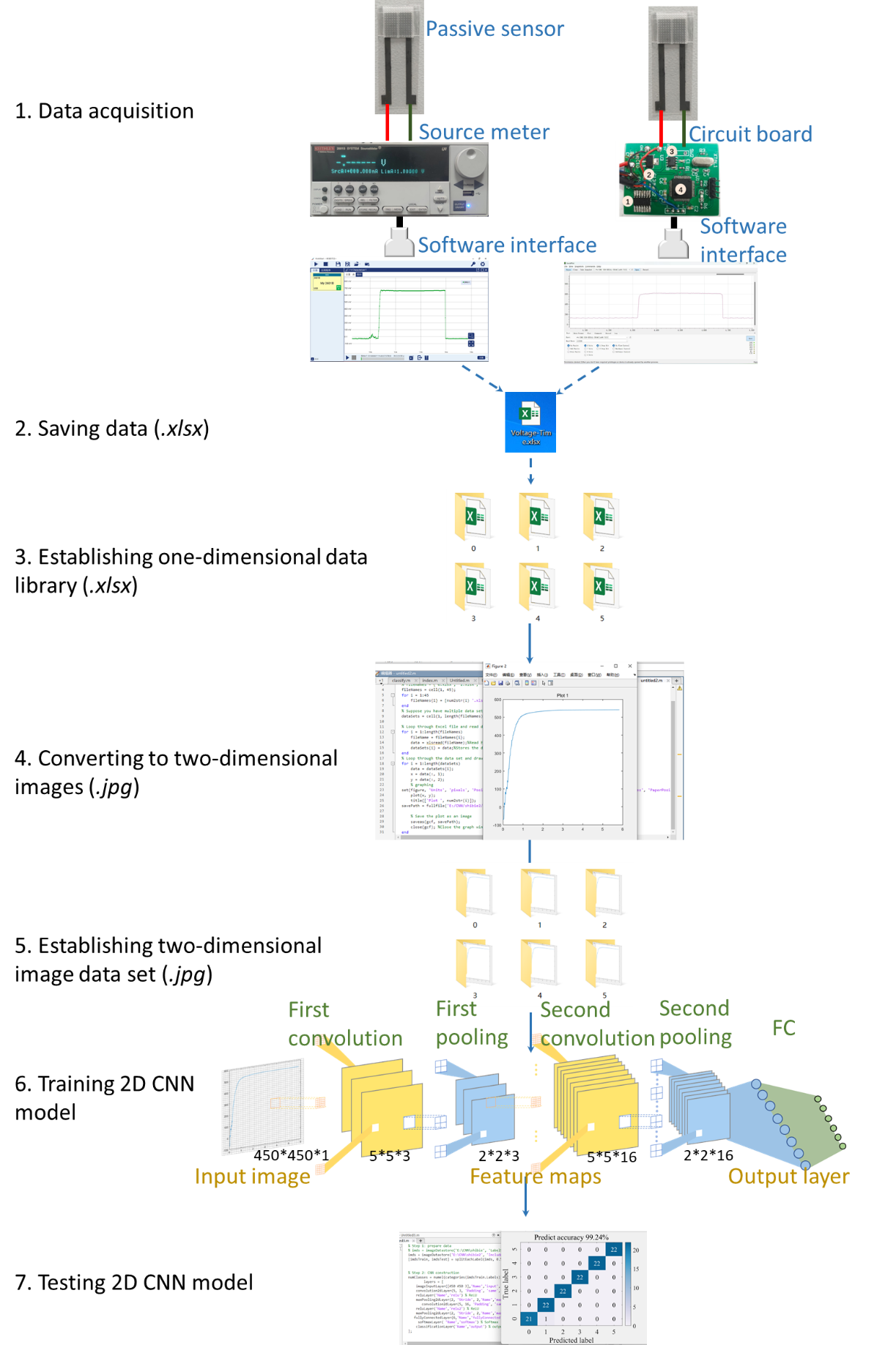
**

**Fig. S3** The full flow chart elaborating the entire data collection and processing procedures for single point tactile perception (namely material property perception) with the assistance of a machine learning framework based on a 2D CNN

**S1.1 Date acquisition**

In this work, two types of data collection setups can be used to acquire the potential difference outputs of the tactile sensors. Firstly, the original sensor signals (i.e., potential difference vs time) could be collected using a Keithley 2601B source meter in a *Voltage Measure-Only* mode. The detailed parameter settings for this mode are described as follow: sourcing zero current and measuring open-circuit voltage, with a 1 V open circuit voltage limit and a 20 ms sampling rate per point, as shown in **Fig. S4**. The continuously measured sensor signals can be displayed in real-time on a computer interface/software or can be saved to a computer terminal through a USB cable.


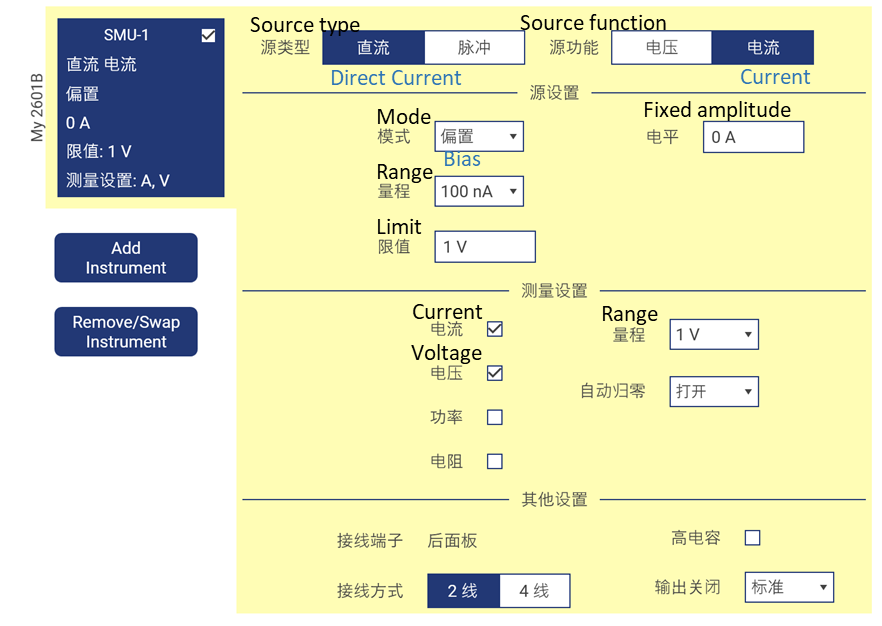


**Fig. S4** A screenshot showing the detailed parameter settings of Keithley 2601B source meter in a *Voltage Measure-Only* mode

Alternatively, the original sensor signals (i.e., potential difference vs time) could also be collected using a custom-made circuit board. The detailed operating principle of the custom-made circuit board is illustrated in **Fig. S5**. The electric schematic diagrams of the circuit board are shown in **Fig. S6**, and the software flowchart and software main code of the circuit board are shown in **Fig. S7**. The potential difference signals generated by the sensors are first transmitted to an operational amplifier with fixed magnification (①). The voltage regular (②) provides steady power supply to ensure the stable operation of the amplifier. After processing, the signals are then transmitted to the STM 32 microprocessor (④) and are centralized for final processing. The analog-to-digital converter of the STM 32 microprocessor is powered by a reference voltage (③). The development environment of stm32 microprocessor acquisition device is Keil, and the software part of the whole system is designed by C language. It mainly includes the writing of the main program, the setting of data transmission, and AD collection. The main program is responsible for the system clock configuration, AD configuration, and GPIO initialization. AD acquisition includes a median filter, which filters a set of AD conversion values with a median average and returns the filtered results. The data collected by the AD is converted into the actual voltage of the sensor and then sent to the PC through the serial port. The measured sensor signals with this circuit board are recorded and presented on a software for visualization and post-processing using a USB interface.


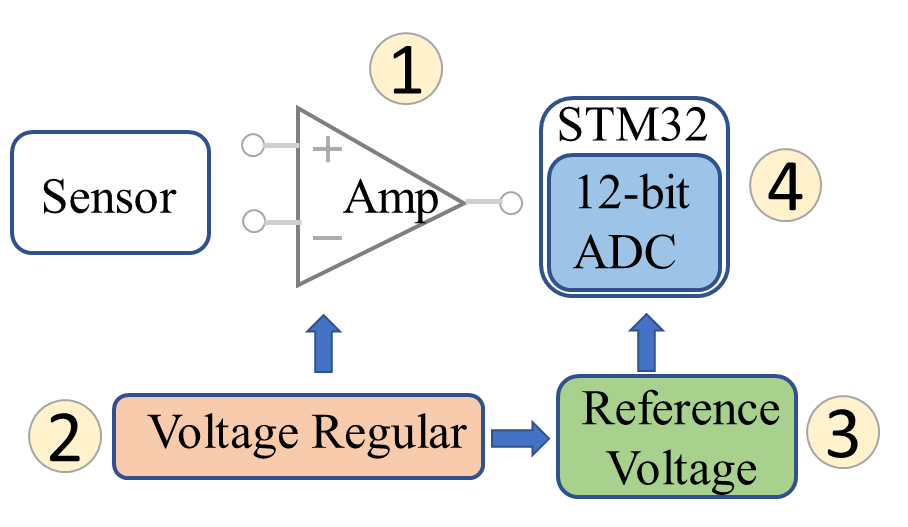

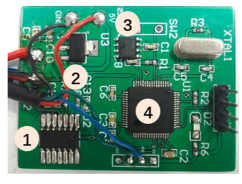


**Fig. S5** Illustration depicting the operating principle of the circuit board and digital picture of the custom-designed circuit board used here for the potential difference measurement

**
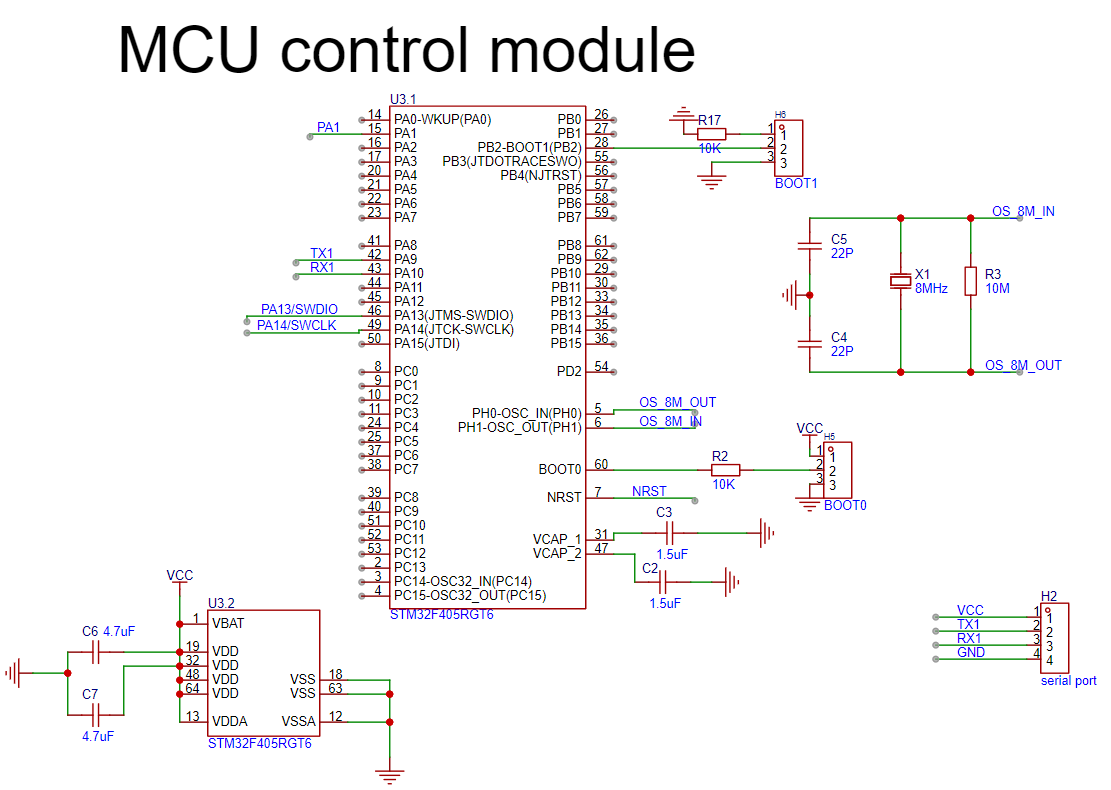

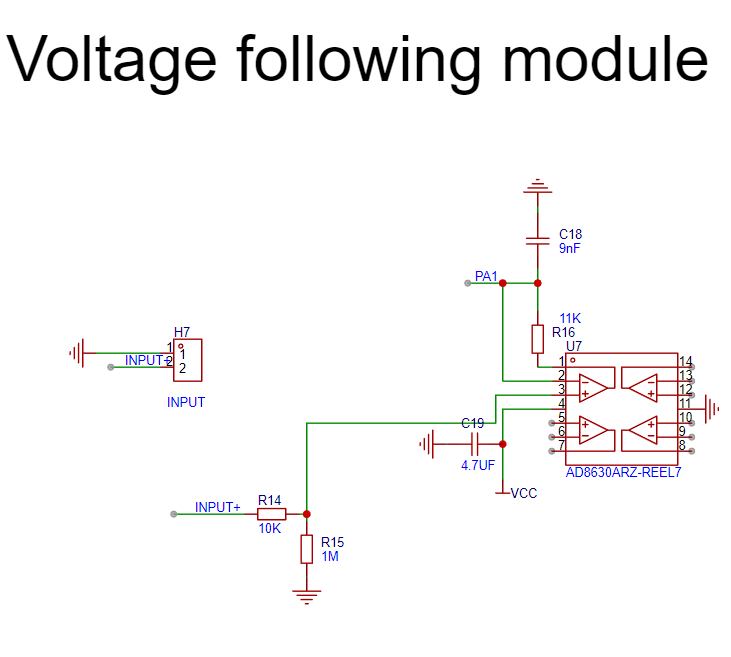
**

**Fig. S6** Electric schematic diagram showing more details of the circuit board.


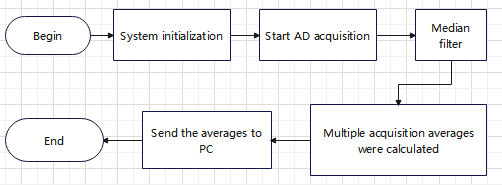
**
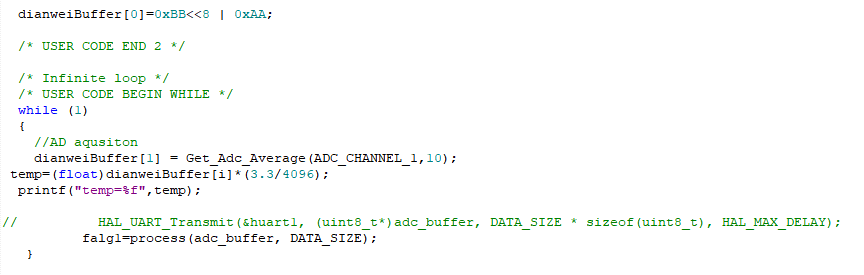

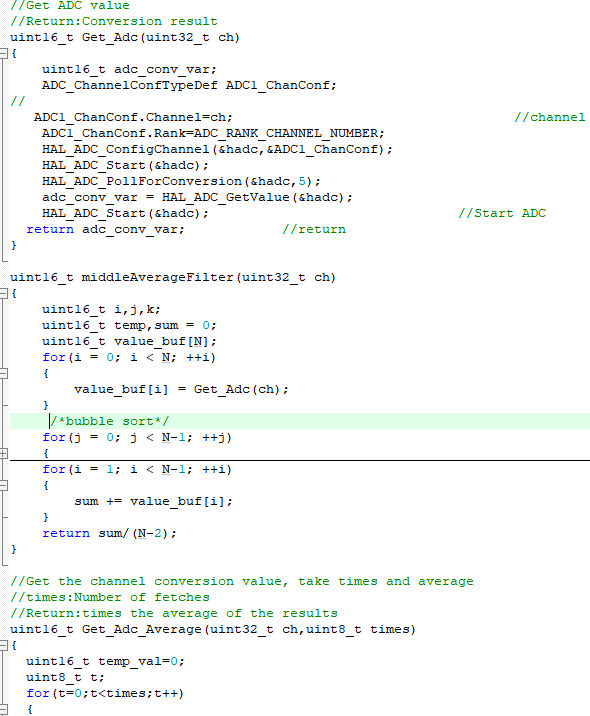
**

**Fig. S7** The software flowchart and software main code used in this work for the potential difference measurement with the circuit board

Through either of the above-mentioned measuring devices, the one-dimensional sensor signals (potential difference vs time) can be collected and displayed in real time. It is worth noting that the date transmission from front-end measuring devices and software interfaces to the post-processing programs (e.g., 2D CNN framework) is not in real time in this work. Specifically, the recorded one-dimensional data needs to be manually exported from the acquisition software into folders to establish one-dimensional data library (as elaborated in **Fig. S3**), then, the one-dimensional data needs to be converted into 2D images with data processing software for the subsequent training and testing of the 2D CNN model.

**S1.2 Date processing**

***1.2.1 Establishing of the two-dimensional image data set***

The program script iteratively accessed the folders 0-5 (which are the one-dimensional sensor signals recorded during touching different objects), followed by processing 45 sets of Excel files within each folder, extracting the columns representing time and voltage, drawing a voltage response curve, and then saving each set of 45 curves as a *.jpg* file in the corresponding folder 0-5 (as shown in **Fig. S8**) to finally establish the two-dimensional data set.

Subsequently, the two-dimensional data set were divided into the training set and the testing set. The training set had around 49% of the 2D image data (22 image data) and the test set had around 51% of the 2D image data (23 image data) in each file. This above-mentioned program simplifies the process of data extraction and visualization, ensuring efficient processing of data sets.


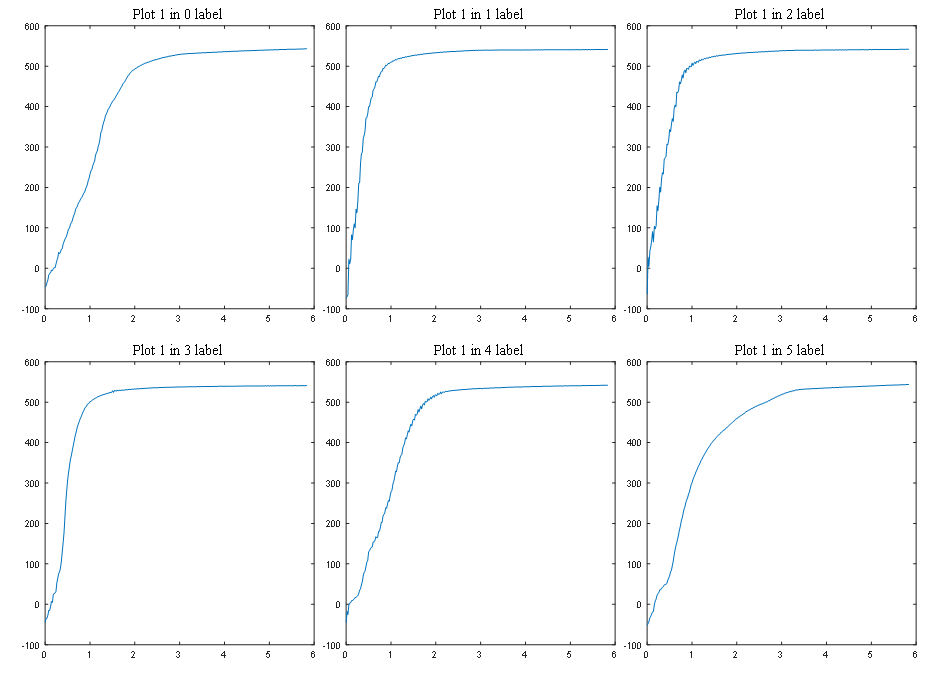


**Fig. S8** Typical examples of the automatically generated two-dimensional images with the program script

***Original Code Used in This Section:***

*%% Automatically draw and save images*

*% Suppose you have multiple Excel files to read, and the file names are stored in an array of cells*

*fileNames = cell(1, 45);*

*for i = 1:45*

*fileNames{i} = [num2str(i) '.xlsx'];*

*end*

*% Suppose you have multiple data sets to draw, stored in an array of cells*

*dataSets = cell(1, length(fileNames)); %Create an empty array of cells to store the data*

*% Loop through Excel file and read data*

*for i = 1:length(fileNames)*

*fileName = fileNames{i};*

*data = xlsread(fileName);%Read Excel data*

*dataSets{i} = data;%Stores the data in the cell array*

*end*

*% Loop through the data set and draw the graph*

*for i = 1:length(dataSets)*

*data = dataSets{i};*

*x = data(:, 1);*

*y = data(:, 2);*

*% graphing*

*set(figure, 'Units', 'pixels', 'Position', [0, 0, 500, 500], 'Renderer', 'painters', 'PaperUnits', 'inches', 'PaperPosition', [0, 0, 3, 3]);*

*plot(x, y);*

*title(['Plot ', num2str(i)]);*

*savePath = fullfile('E:/CNN/6', ['plot', num2str(i), '.jpg']);%Change the storage location manually*

*% Save the plot as an image*

*saveas(gcf, savePath);*

*close(gcf); %Close the graph window so that it reopens on the next iteration*

*end*

*imds = imageDatastore('D:\CNN\classify', 'IncludeSubfolders', true, 'LabelSource', 'foldernames');*

*[imdsTrain, imdsTest] = splitEachLabel(imds, 0.5, 'randomize');*

*labelCount = countEachLabel(imds)*

***S1.2.2 Classification via 2D CNN***

In the family of image processing methods, CNN has become one of the most commonly used and effective models for image processing. By training CNN models, automatic image classification with relatively high accuracy can be achieved. In this work, we also employed 2D CNN models to process and classify the tactile sensor signals.

***1.2.2.1 Establishing of the 2D CNN model***

***The input layers***：The input layers of the 2D CNN model used in this work were potential difference variation curve images with the size of 450×450.

***The first convolution layers***：The first convolution layers contained 3 convolution kernels with the size of 5×5.

***The first max pooling layers***：The first max pooling layers had 2×2 pooled core, and the feature map was pooled by moving a window with a step of 2.

***The second convolution layers***：The second convolution layers contained 16 convolution kernels with the size of 5×5.

***The second max pooling layers***：The second max pooling layers had 2×2 pooled core, and the feature map was pooled by moving a window with a step of 2.

***Fully connected layers***：Fully connected layers had 6×200704 neurons. Each neuron in this layer will be connected to all the neurons in the previous layer.

***The output layers***：The output layers comprise 6 neurons that symbolized the types of texture types that needs to be recognized.

The 2D CNN model is composed of two convolution layers with ReLUs activation. The convolution layers are set to use the ‘same’ padding method, so they do not change the size of the data. By using max pooling on the feature maps in the convolution layers, the output size was reduced to half. After two consecutive convolution and max pooling operations, we could obtain a three-dimensional vector array of size 112×112×16, where 16 was the number of channels. After a fully connected layers activated by softmax, the size of the data became 1×1×6, as shown in **Fig. S9**.


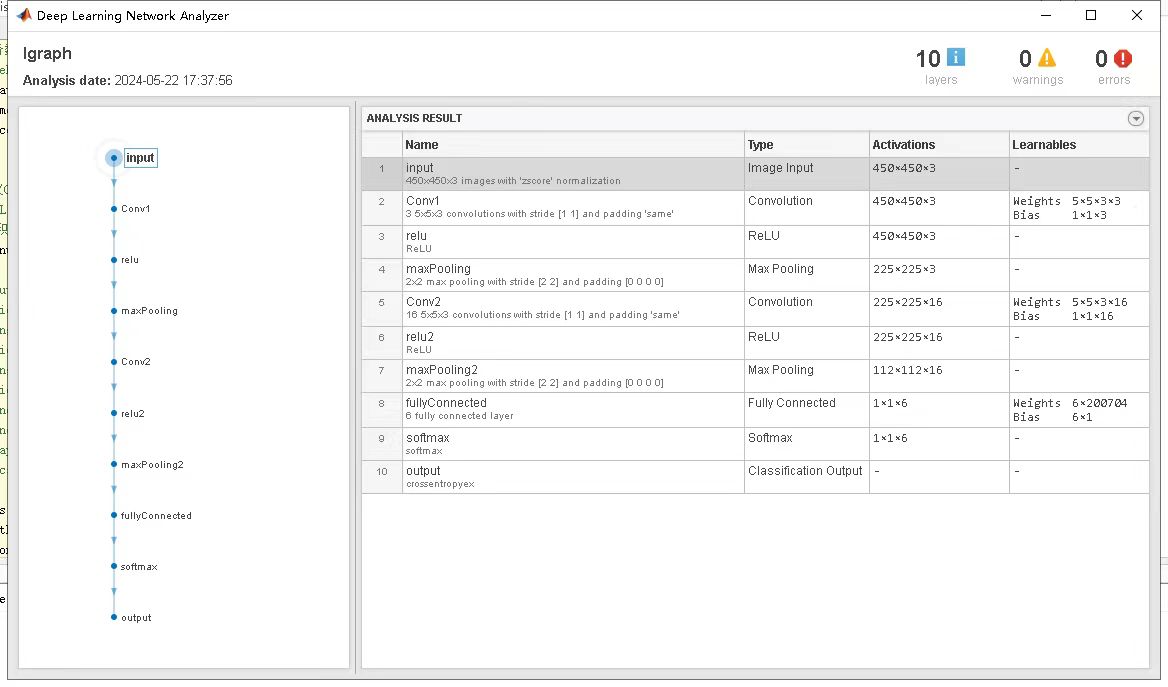


**Fig. S9** The data size change of 2D CNN model

***S1.2.2.2 Training of the 2D CNN model***

Parameter optimization was carried out using Stochastic Gradient Descent (SGD) with an initial learning rate of lr=0.001 and a maximum training epoch of 8. Validation was conducted every 30 mini-batches, and the mini-batch size was set to 6. The network was trained using the data from the training set. The 2D CNN model training process is shown in **Fig S10**.


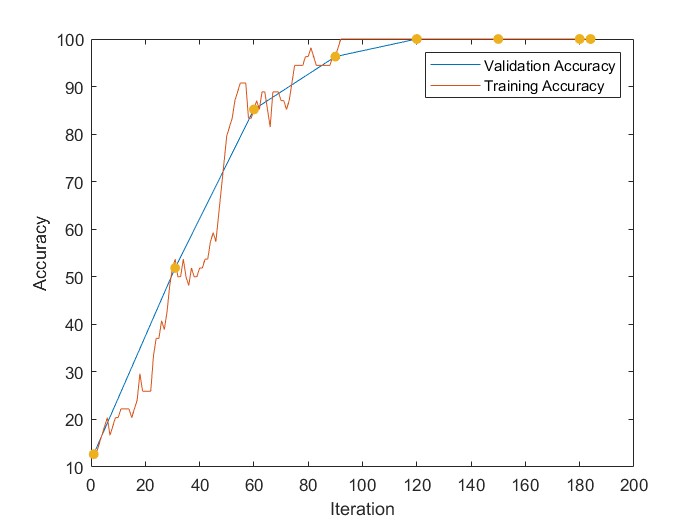

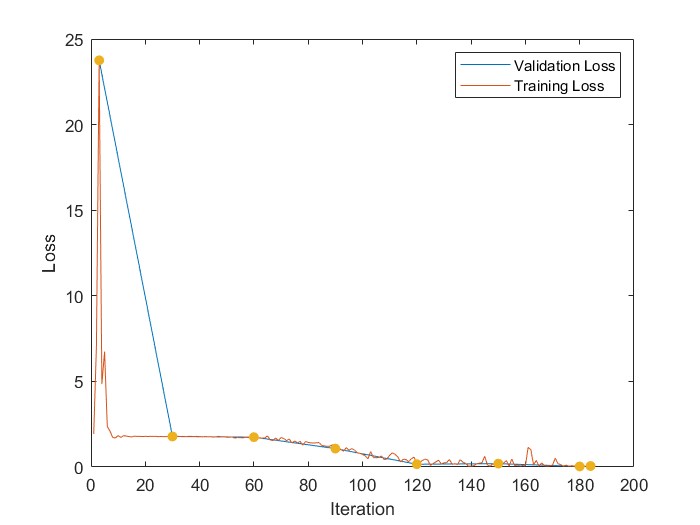


**Fig. S10** The accuracy and loss curves of the 2D CNN training process

***S1.2.2.3 Testing of the 2D CNN model***

The trained neural network was used to make classification prediction for the test data set, and the prediction results were stored in prediction labels. The predicted labels were compared with the real labels to calculate the classification accuracy, and finally, the confusion matrix was plotted.

***Original Code Used in This Section:***

*% Defining the CNN Architecture*

*numClasses = numel(categories(imdsTrain.Labels));*

*layers = [*

*imageInputLayer([450 450 3],'Name','input','Normalization','zscore') %Input layer, assuming image size is, number of channels is 3 (color image)*

*convolution2dLayer(5, 3, 'Padding', 'same','Name','Conv1') % convolutional layer*

*reluLayer('Name','relu') % ReLU Activation function layer*

*maxPooling2dLayer(2, 'Stride', 2,'Name','maxPooling') % Maximum pooling layer*

*convolution2dLayer(5, 16, 'Padding', 'same','Name','Conv2') % convolutional layer*

*reluLayer('Name','relu2') % ReLU Activation function layer*

*maxPooling2dLayer(2, 'Stride', 2,'Name','maxPooling2') % Maximum pooling layer*

*fullyConnectedLayer(6,'Name','fullyConnected') % Fully connected layer*

*softmaxLayer( 'Name','softmax') % Softmax layer*

*classificationLayer('Name','output') % classification layer*

*];*

*options = trainingOptions('sgdm', ...*

*'InitialLearnRate',0.001, ...*

*'MaxEpochs',8, ...*

*'Shuffle','every-epoch', ...*

*'ValidationData',imdsTrain, ...*

*'ValidationFrequency',30, ...*

*'Verbose',true, ...*

*'MiniBatchSize',6, ...*

*'Plots','training-progress');*

*% train*

*net = trainNetwork(imdsTrain,layers,options);*

*predLabels = classify(net, imdsTest, 'MiniBatchSize', 42)*

*% calculate accuracy*

*accuracy = mean(predLabels == imdsTest.Labels);*

*% calculate confusionmat*

*C = confusionmat(imdsTest.Labels, predLabels);*

*function confusion_matrix_plot(mat,a)*

*is_normalize=false;*

*mat = fliplr(mat);*

*if is_normalize*

*mat=mat./(sum(mat,2));*

*end*

*if size(mat,1)~=size(mat,2)*

*error("Error occurred: Wrong input!")*

*end*

*dim=size(mat,1);*

*imagesc(mat);*

*colormap(othercolor('PuBu6'));*

*brighten(0.1)*

*if ~is_normalize*

*textStrings = num2str(mat(:),'%d');*

*else*

*textStrings = num2str(mat(:).*100,'%.2f');*

*textStrings=strcat(textStrings,{'%'});*

*end*

*textStrings = strtrim(cellstr(textStrings));*

*[x,y] = meshgrid(1:dim);*

*hStrings = text(x(:),y(:),textStrings(:),'HorizontalAlignment',...*

*'center','FontSize',18,'Fontname', 'Times New Roman');*

*textColors = repmat(mat(:) > mean(get(gca,'CLim')),1,3);*

*set(hStrings,{'Color'},num2cell(textColors,2));*

*set(gca,'xtick',1:dim,...*

*'xticklabel',{'0','1','2','3','4','5'},...*

*'XTickLabelRotation',0,...*

*...*

*'ytick',1:dim,...*

*'yticklabel',{'5','4','3','2','1','0'},...*

*'YTickLabelRotation',90,...*

*'FontSize',18,'Fontname', 'Times New Roman');*

*title(['Predict accuracy ' num2str(a) '%']);*

*ylabel('True label');*

*xlabel('Predicted label');*

*colorbar;*

*end*


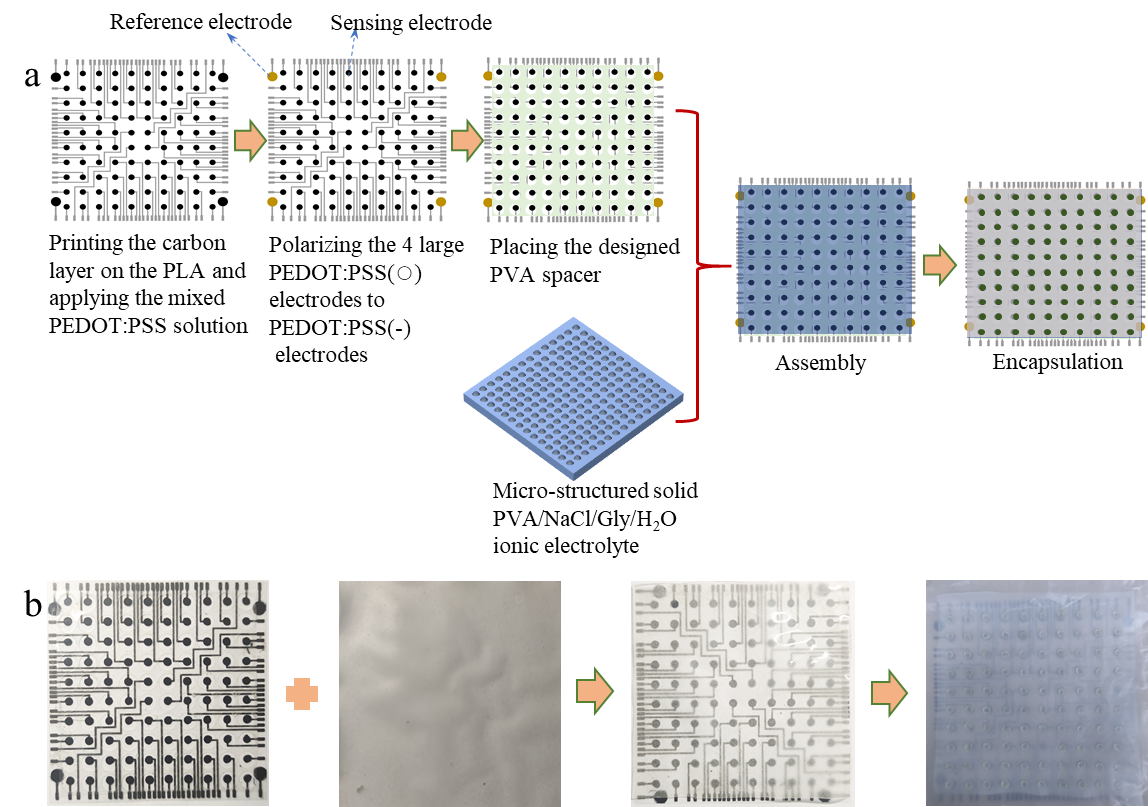


**Fig. S11** Schematic illustrations (**a**) and photographs (**b**) showing the fabrication of the single-electrode-mode e-skin

**Note S2 Data collection and processing methods for two-dimensional tactile sensation**

**
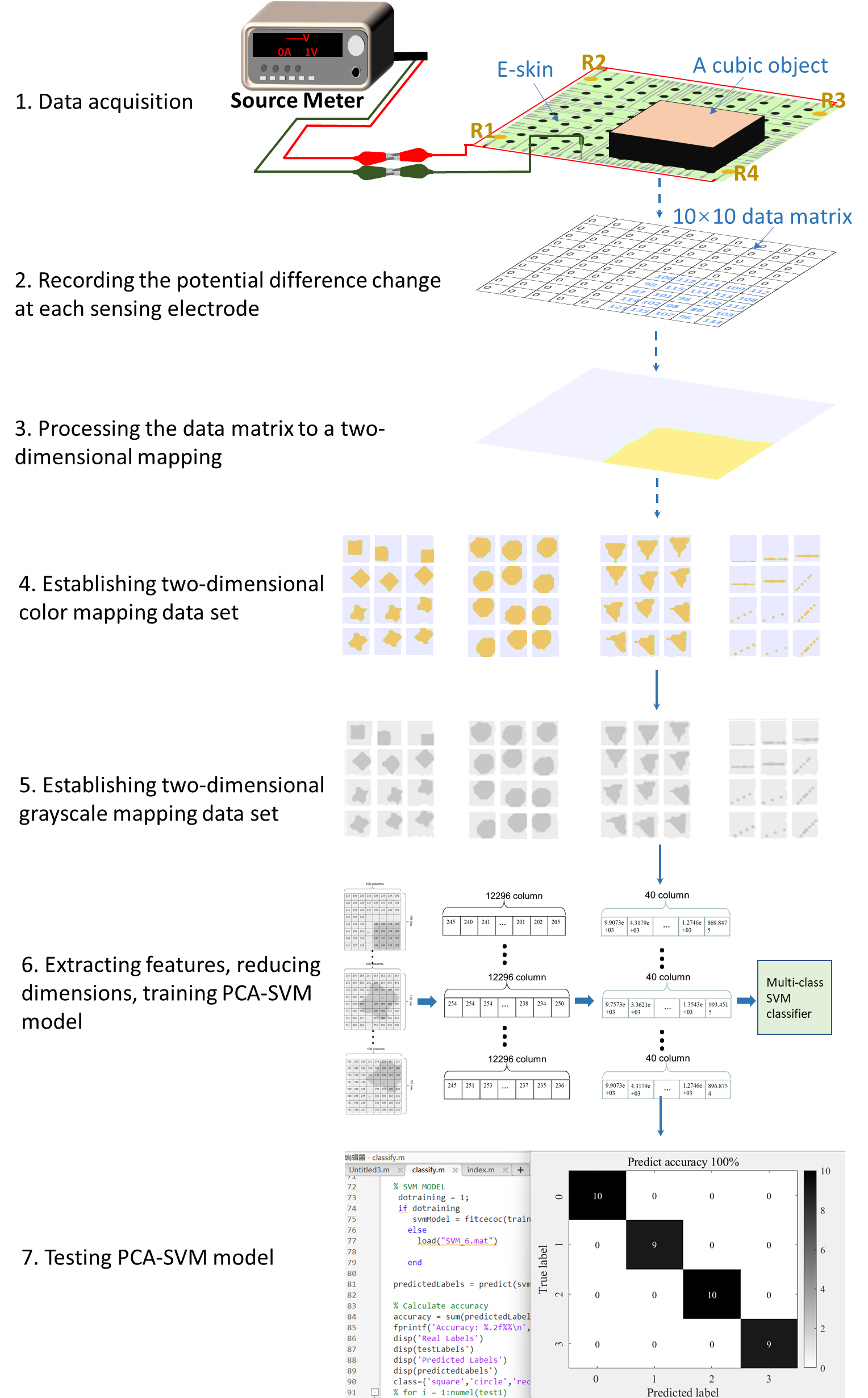
**

**Fig. S12** The full flow chart elaborating the entire data collection and processing for the two-dimensional tactile sensation (i.e., object shape or profile perception) with the assistance of a machine learning framework based on PCA-SVM model

**S2.1 Date acquisition**

The passive e-skin includes 10*10 sensing electrodes (labeled S1 to S100) and four reference electrodes (labeled R1 to R4). When an object was pressed on the electronic skin, the potential difference outputs measured between each of the sensing electrodes and the reference electrodes of the electronic skin was recorded one by one, and not measured simultaneously. A source meter (Keithley 2601B) with one measuring channel was used to measure the potential difference outputs, and the parameter settings are detailed above (see Note S1). The four reference electrodes (R1-R4) were simultaneously connected to the reference electrode channel of the source meter to improve the accuracy due to the different distances between the sensing electrodes and the reference electrodes. Then, the working electrode channel of the source meter was successively connected to the sensing electrodes of the e-skin (from S1 to S100, one by one). The corresponding potential difference outputs from all of the sensing electrodes were manually recorded in a 10*10 table.

**S2.2 Date processing**

***S2.2.1 Establishing of the two-dimensional color mapping data set***

The recorded original data matrix (i.e., 10*10 data set) were imported into mapping software (e.g., Origin) to construct the two-dimensional color mapping data set.

***S2.2.2 Conversion into two-dimensional grayscale mapping data set with the written control program***

The four different color mapping data set from four different objects (including cube, cylinder, triangular prism, and rod) were converted into four different grayscale mapping data set and stored in four different folder paths.

***Original Code Used in This Section:***

*Img = imread("img.png");*

*grayImg = rgb2gray(Img); %Grey Processing*

*for i = 1:12 %Categorize and store image data*

*Img1 = grayImg(1:116, 6+105*(i-1):7+105*(i-1) + 104);*

*imgname = sprintf("./square/img%d.jpg", i);*

*imwrite(Img1, imgname);*

*end*

*for i = 1:12*

*Img1 = grayImg(117:232, 6+105*(i-1):7+105*(i-1) + 104);*

*imgname = sprintf("./circle/img%d.jpg", i);*

*imwrite(Img1, imgname);*

*end*

*for i = 1:12*

*Img1 = grayImg(233:348, 6+105*(i-1):7+105*(i-1) + 104);*

*imgname = sprintf("./triangle/img%d.jpg", i);*

*imwrite(Img1, imgname);*

*end*

*for i = 1:12*

*Img1 = grayImg(349:464, 6+105*(i-1):7+105*(i-1) + 104);*

*imgname = sprintf("./rectangle/img%d.jpg", i);*

*imwrite(Img1, imgname);*

*end*

***S2.2.3 Classification via PCA-SVM***

***S2.2.3.1 Extracting features***

Since the images are grayscale mappings, the values of the mapping data are between 0 and 255. The mapping data under different folder paths were read iteratively, and the image data were stored in the *i*^th^ row and the first column (48 rows in total) of the data cell array. The corresponding class labels of the image ( including “0”, “1”, “2”, and “3”) were stored in row *i*^th^ and the second column of the data cell array. The data sets associated with each mapping sample and their corresponding class labels were obtained.

***S2.2.3.2 PCA dimensional reduction***

The employment of the PCA method for dimensional reduction of the mapping data are described as follow and shown in **Fig 13**:

***Step1***: The number of features of each grayscale mapping was calculated as 12296 by the size of image data 116*106.

***Step2***: The images were reshaped into a single row vector and then stored in row i of a feature matrix of size 48 *12296.

***Step3***: The principal component loading coefficients were calculated from the feature matrix and the coefficients were stored in the coefficient matrix. The order of the coefficients in the matrix is based on their ability to explain the variance of the data from high to low.

***Step4***: The coefficient matrix of the specified dimension (40) with the feature matrix was multiplied to obtain the PCA feature matrix, which was used for dimensionality reduction. The 48 vectors after dimensionality reduction were used as the data set.


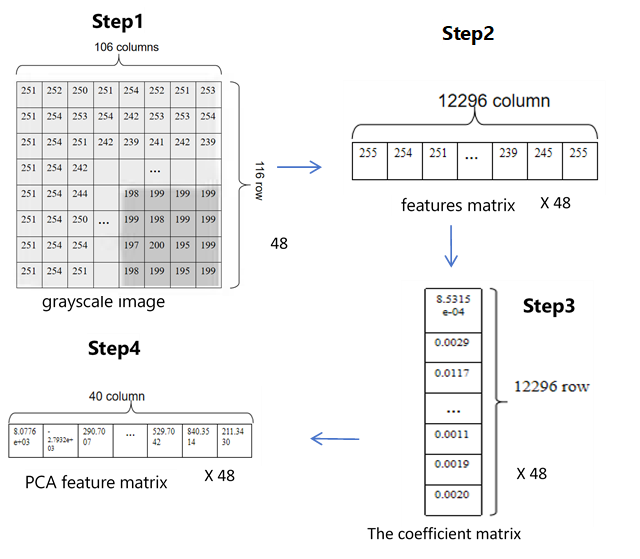


**Fig. S13** The flow diagram showing the PCA dimensional reduction process

***S2.2.3.3 Training of the PCA-SVM model***

Since support vector machine (SVM) is a binary classifier, multi-class classification is realized by using ECOC method. ECOC is a code matrix composed of {-1,0,1}, set as M, where the rows of M correspond to the number of sample classes and the columns correspond to the number of classifiers, and the multiple classification is decomposed into 6 binary classification problems, as shown in **Fig. S14**.


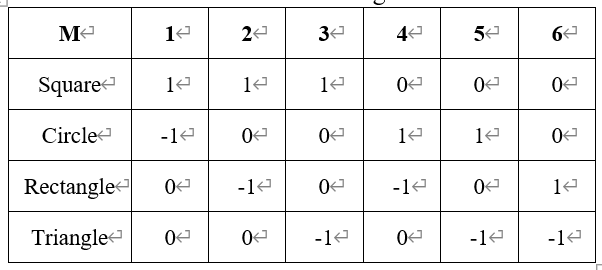


**Fig. S14** ECOC for the 4 categories of object classification

The data was divided into a training set and a testing set. The multi-class SVM classifier used the training set to get the trained SVM model, which then can be used to classify and predict the testing data, as shown in **Fig. S15**.


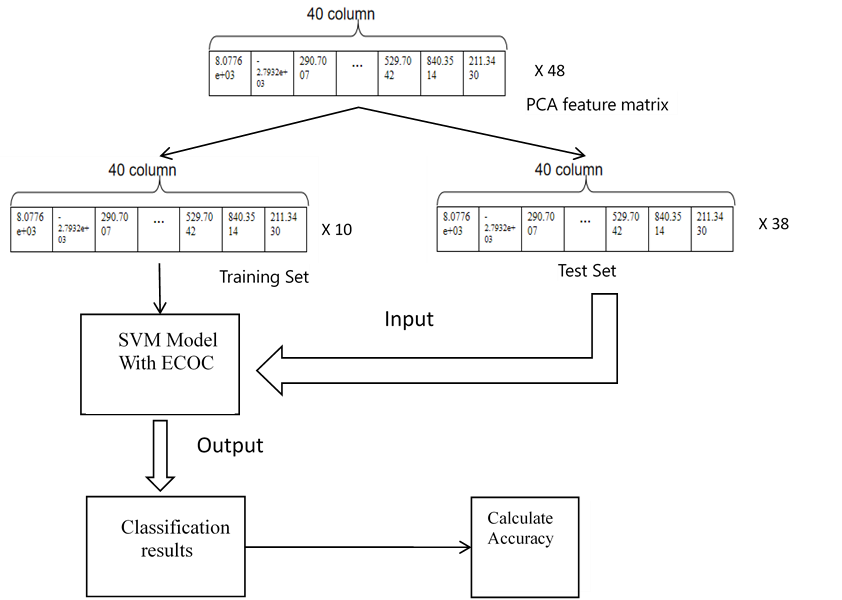


**Fig. S15** The diagram showing the training process of the PCA-SVM model

***S2.2.3.4 Test of the PCA-SVM model***

The trained PCA-SVM model was used on the testing data set to obtain the prediction label, and the quality of the model was evaluated by comparing the calculation accuracy of the prediction label with the real label. The proportion of testing sets increased from 0.2 to 0.8, and the classification accuracy of the model remained a relative high level, which indicated the reliability of the PCA-SVM model.

***Original Code Used in This Section:***

*% Define folder path*

*folderPath1 = './image/square/';*

*folderPath2 = './image/circle/';*

*folderPath3 = './image/rectangle/';*

*folderPath4 = './image/triangle/';*

*data = cell(48, 2);*

*files = dir(fullfile(folderPath1, '*.jpg'));*

*for i = 1:size(files, 1)*

*filePath = fullfile(folderPath1, files(i).name);*

*data{i, 1} = imread(filePath);*

*data{i, 2} = 0;*

*end*

*files = dir(fullfile(folderPath2, '*.jpg'));*

*for i = 1:size(files, 1)*

*filePath = fullfile(folderPath2, files(i).name);*

*data{i+12, 1} = imread(filePath);*

*data{i+12, 2} = 1;*

*end*

*files = dir(fullfile(folderPath3, '*.jpg'));*

*for i = 1:size(files, 1)*

*filePath = fullfile(folderPath3, files(i).name);*

*data{i+24, 1} = imread(filePath);*

*data{i+24, 2} = 2;*

*end*

*files = dir(fullfile(folderPath4, '*.jpg'));*

*for i = 1:size(files, 1)*

*filePath = fullfile(folderPath4, files(i).name);*

*data{i+36, 1} = imread(filePath);*

*data{i+36, 2} = 3;*

*end*

*% shuffle label and data*

*indices = randperm(size(data, 1));*

*data = data(indices, :);*

*% claculate feature*

*numFeatures = size(data{1, 1}, 1) * size(data{1, 1}, 2);*

*features = zeros(size(data, 1), numFeatures);*

*for i = 1:size(data, 1)*

*img = data{i, 1};*

*features(i, :) = img(:)';*

*end*

*numDimensions = 40;*

*coeff = pca(features);*

*pcaFeatures = features * coeff(:, 1:numDimensions);*

*numSamples = size(data, 1);*

*trainRatio = 0.2;*

*trainSize = round(numSamples * trainRatio);*

*trainData = pcaFeatures(1:trainSize, :);*

*test=data(trainSize+1:size(data),:);*

*test1=test(:,1);*

*trainLabels = cell2mat(data(1:trainSize, 2));*

*testData =pcaFeatures(trainSize+1:end, :);*

*testLabels = cell2mat(data(trainSize+1:end, 2));*

*% SVM MODEL*

*dotraining =0;*

*if dotraining==0*

*svmModel = fitcecoc(trainData, trainLabels);*

*dotraining=dotraining+1;*

*else*

*load("SVM_7.mat")*

*end*

*predictedLabels = predict(svmModel, testData);*

*% Calculate accuracy*

*accuracy = sum(predictedLabels == testLabels) / numel(testLabels);*

*fprintf('Accuracy: %.2f%%\n', accuracy * 100);*

*disp('Real Labels')*

*disp(testLabels')*

*disp('Predicted Labels')*

*disp(predictedLabels')*

*C = confusionmat(testLabels, predictedLabels);*

*accuracy=accuracy*100;*

*roundedNum = round((accuracy * 10000)) / 10000;*

*confusion_matrix_plot(C,roundedNum)*

*function confusion_matrix_plot(mat,a)*

*is_normalize=false;*

*if is_normalize*

*mat=mat./(sum(mat,2));*

*end*

*if size(mat,1)~=size(mat,2)*

*error("Error occurred: Wrong input!")*

*end*

*dim=size(mat,1);*

*imagesc(mat);*

*colormap(flipud(gray));*

*if ~is_normalize*

*textStrings = num2str(mat(:),'%d');*

*else*

*textStrings = num2str(mat(:).*100,'%.2f');*

*textStrings=strcat(textStrings,{'%'});*

*end*

*textStrings = strtrim(cellstr(textStrings));*

*[x,y] = meshgrid(1:dim);*

*hStrings = text(x(:),y(:),textStrings(:),'HorizontalAlignment',...*

*'center','FontSize',12,'Fontname', 'Times New Roman');*

*textColors = repmat(mat(:) > mean(get(gca,'CLim')),1,3);*

*set(hStrings,{'Color'},num2cell(textColors,2));*

*set(gca,'xtick',1:dim,...*

*'xticklabel',{'0 ','1','2','3'},...*

*...*

*'ytick',1:dim,...*

*'yticklabel',{'0 ','1','2','3'},...*

*'YTickLabelRotation',90,...*

*'FontSize',14,'Fontname', 'Times New Roman');*

*title(['Predict accuracy ' num2str(a) '%']);*

*ylabel('True label');*

*xlabel('Predicted label');*

*colorbar;*

*end*


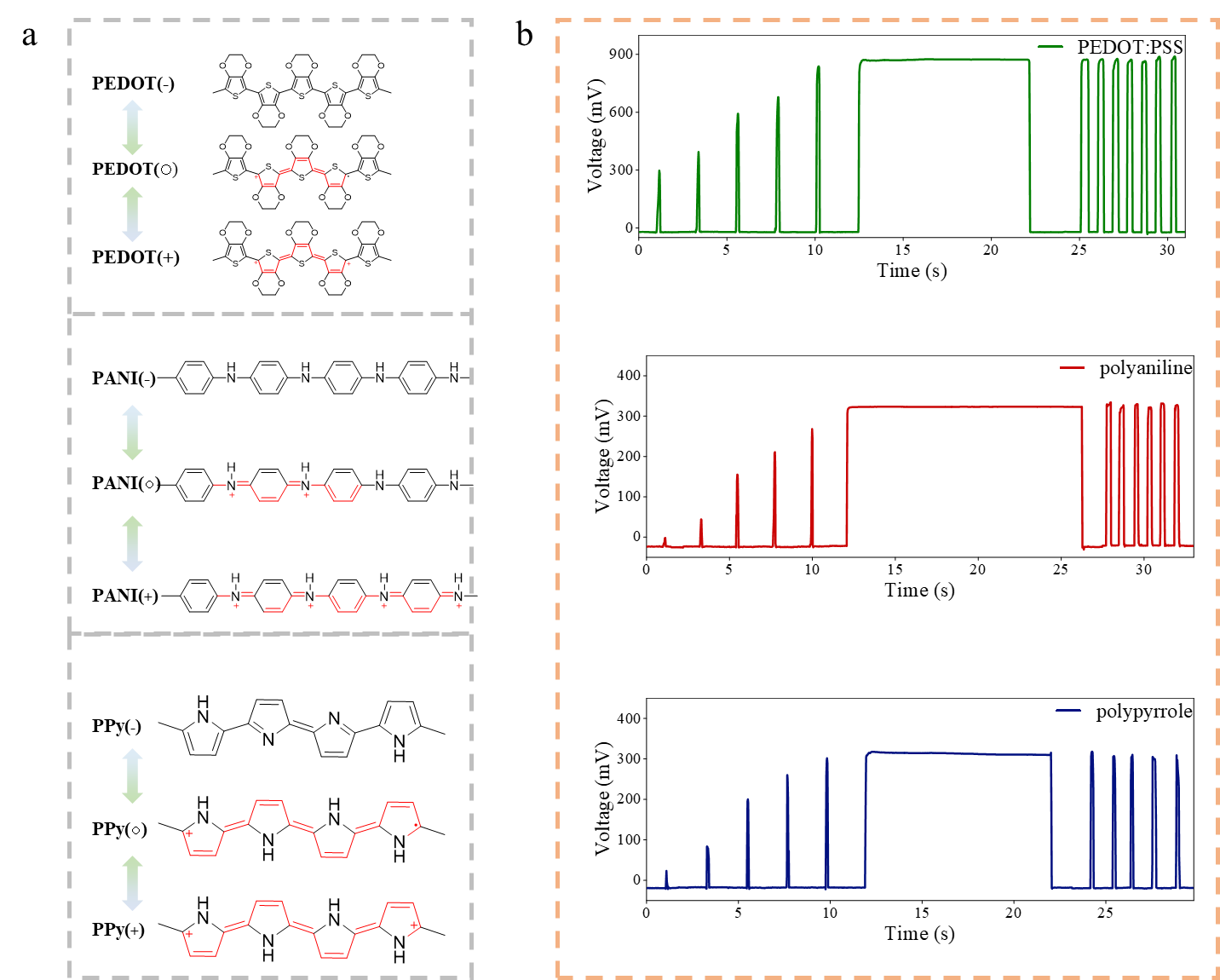


**Fig. S16** (**a**) The structures of PEDOT:PSS, PANI, and PPy before and after polarization. (**b**) Response behaviors of passive tactile sensors constructed from PEDOT:PSS, PANI, and PPy when they are subjected to static and dynamic stimuli of different magnitudes


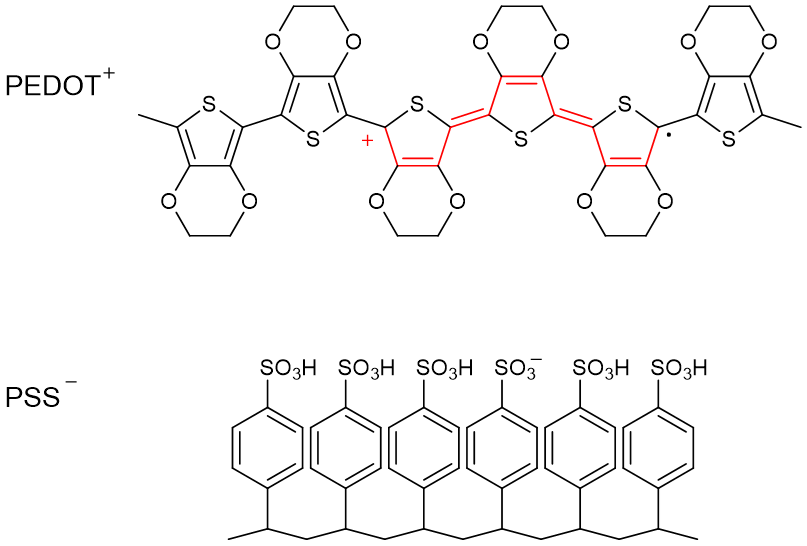


**Fig. S17** Molecular structure of PEDOT:PSS, consisting of conjugated PEDOT backbone and dopant PSS side chains [S1]


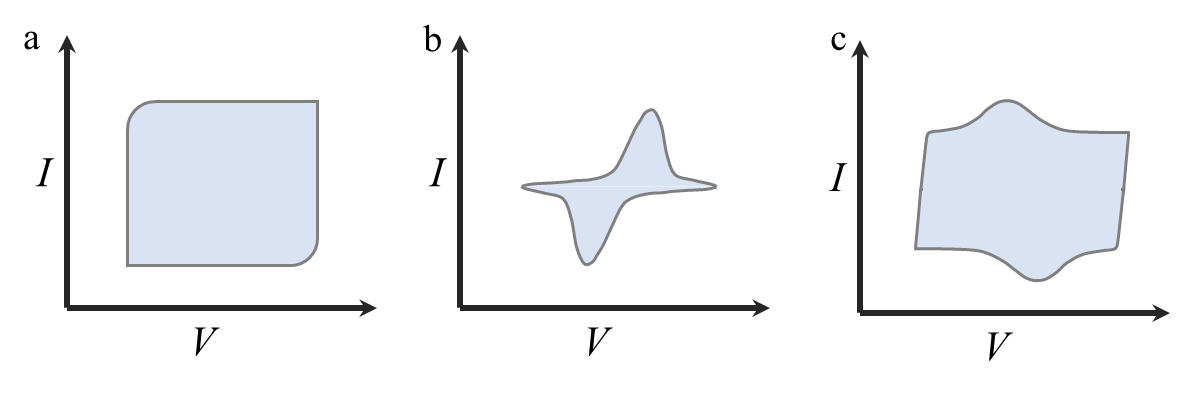


**Fig. S18** Illustrations showing the CV curves from capacitive processes (**a**), Faradaic processes (**b**), and capacitive-Faradaic hybrid processes (**c**) [S2-S4]
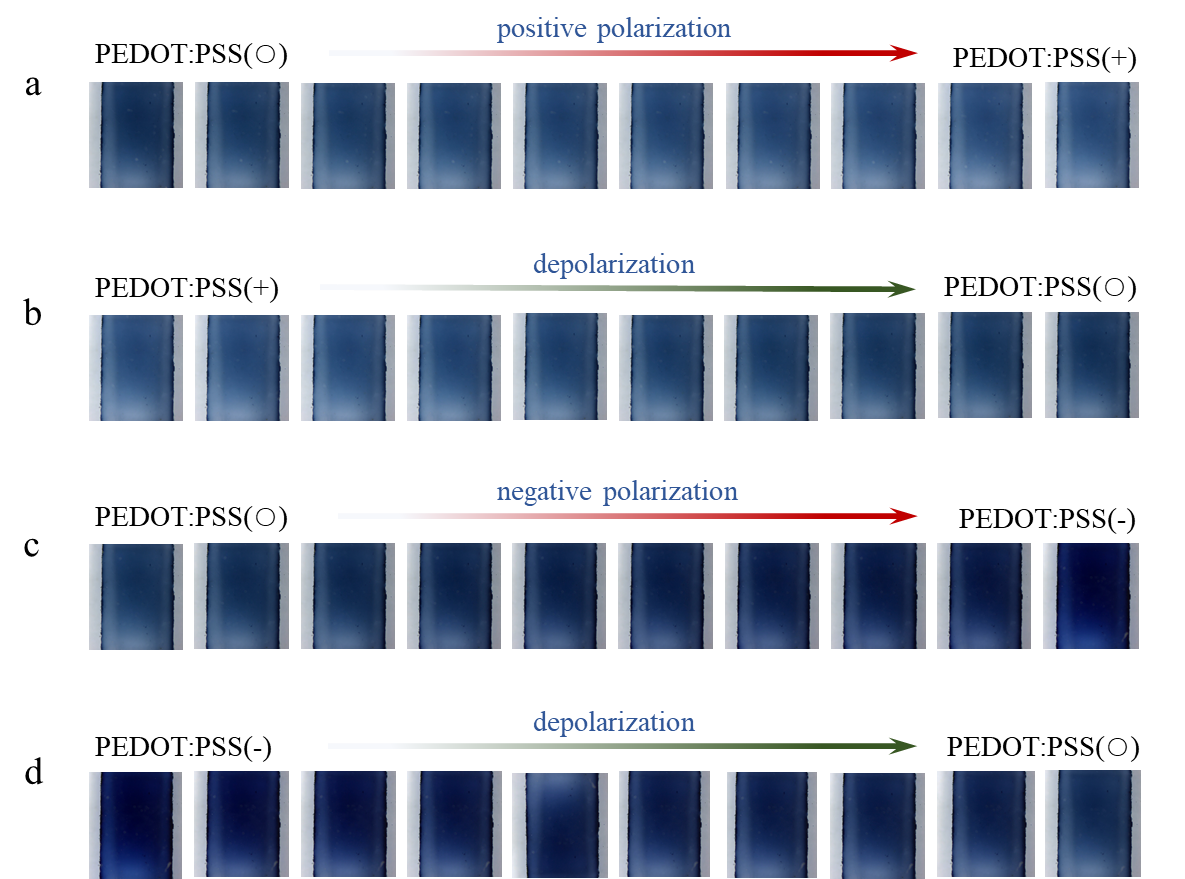


**Fig. S19** In-situ and continuous color change observations from PEDOT:PSS(○) to PEDOT:PSS(+) (**a**). In-situ and continuous color change observations from PEDOT:PSS(+) back to PEDOT:PSS(○) (**b**). In-situ and continuous color change observations from PEDOT:PSS(○) to PEDOT:PSS(-) (**c**). In-situ and continuous color change observations from PEDOT:PSS(-) back to PEDOT:PSS(○) (**d**)


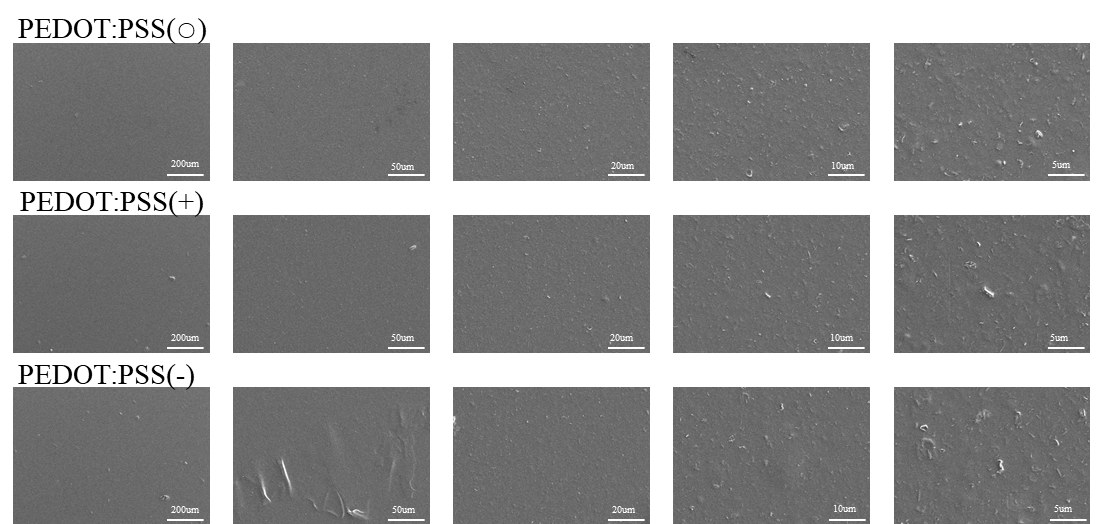


**Fig. S20** SEM images showing the surface morphologies of PEDOT:PSS(○), PEDOT:PSS(+), and PEDOT:PSS(-). Scale bars, 200 µm, 50 µm, 20 µm, 10 µm, and 5 µm, respectively


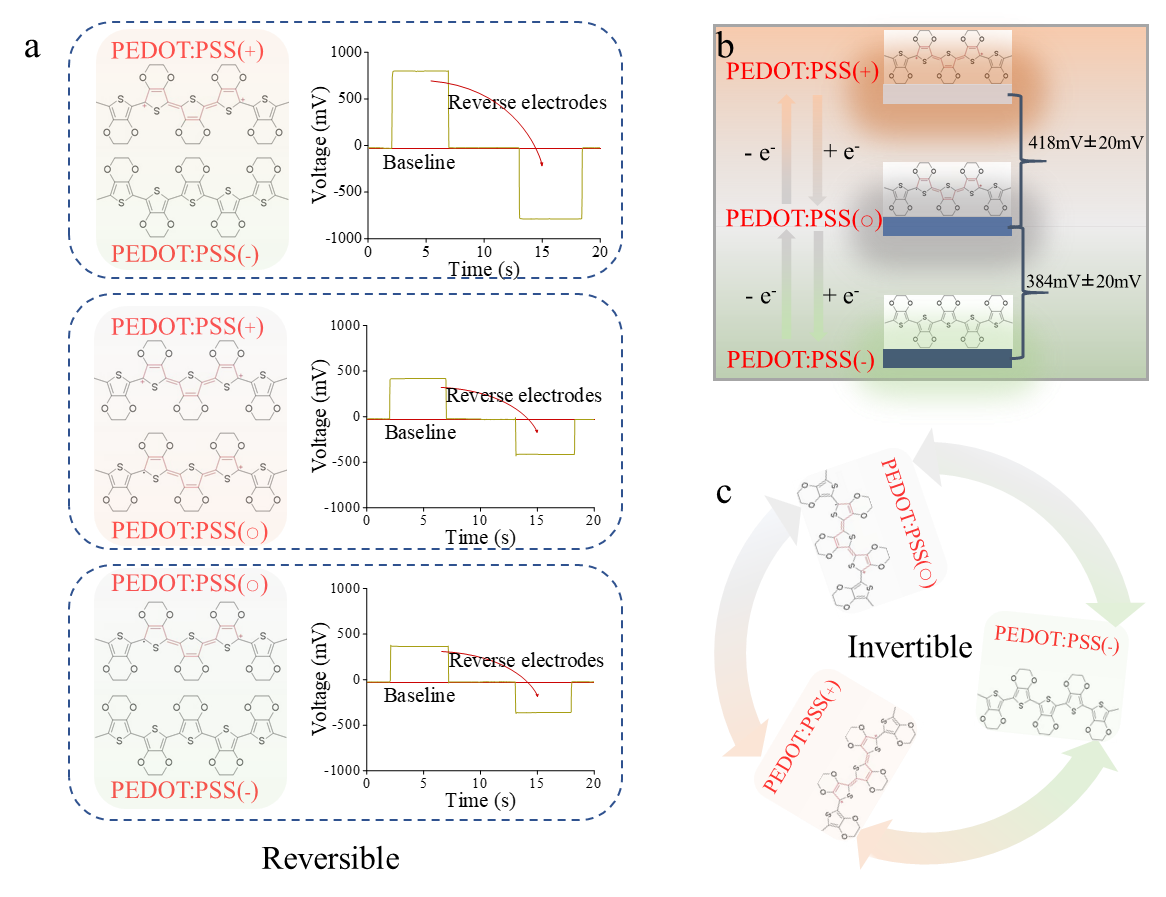


**Fig. S21** Potential difference outputs of different values created between arbitrary combinations of PEDOT:PSS(○), PEDOT:PSS(+), and PEDOT:PSS(-) (**a-b**). Interconversion processes of PEDOT:PSS(○), PEDOT:PSS(+), and PEDOT:PSS(-) by controllable polarization and depolarization (**c**)


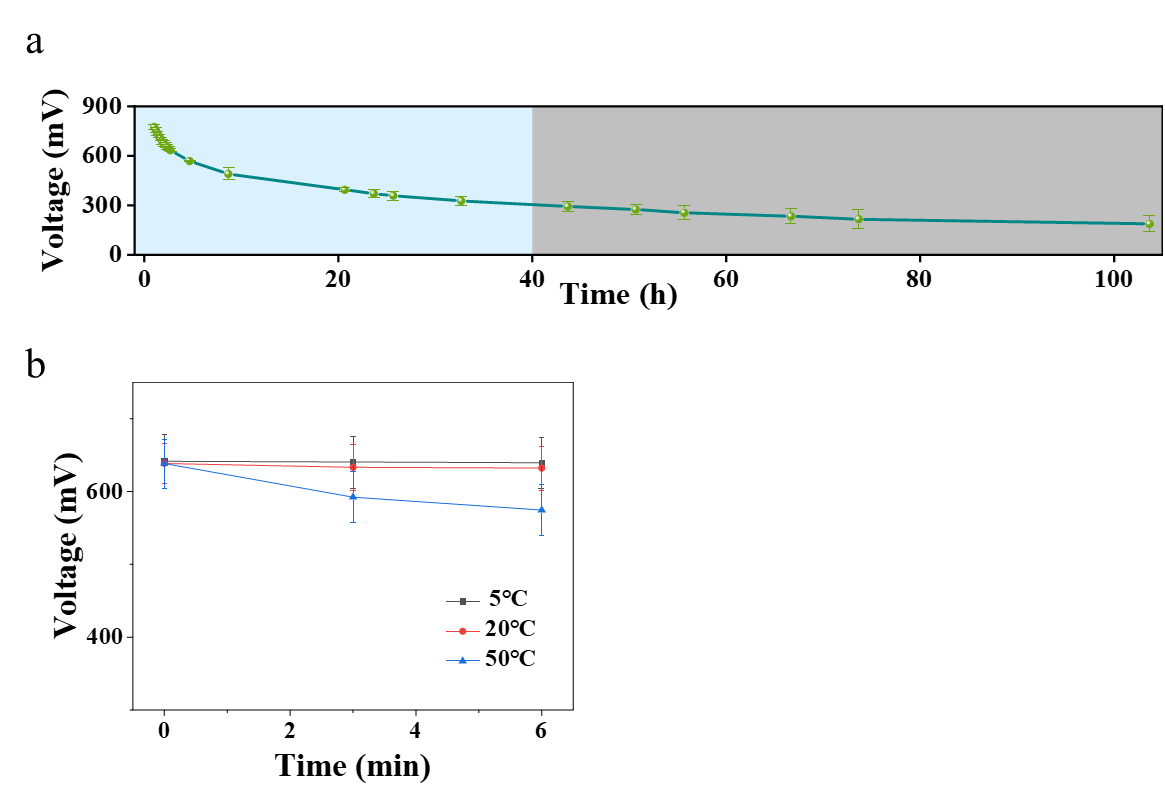


**Fig. S22** (**a**) Potential difference variation measured between PEDOT:PSS(-) and PEDOT:PSS(+) electrodes over a long period of time. (**b**) Potential difference variation of the sensor when the polarized PEDOT:PSS(-) and PEDOT:PSS(+) electrodes were subjected to temperatures of 5℃, 20℃ and 50℃, respectively


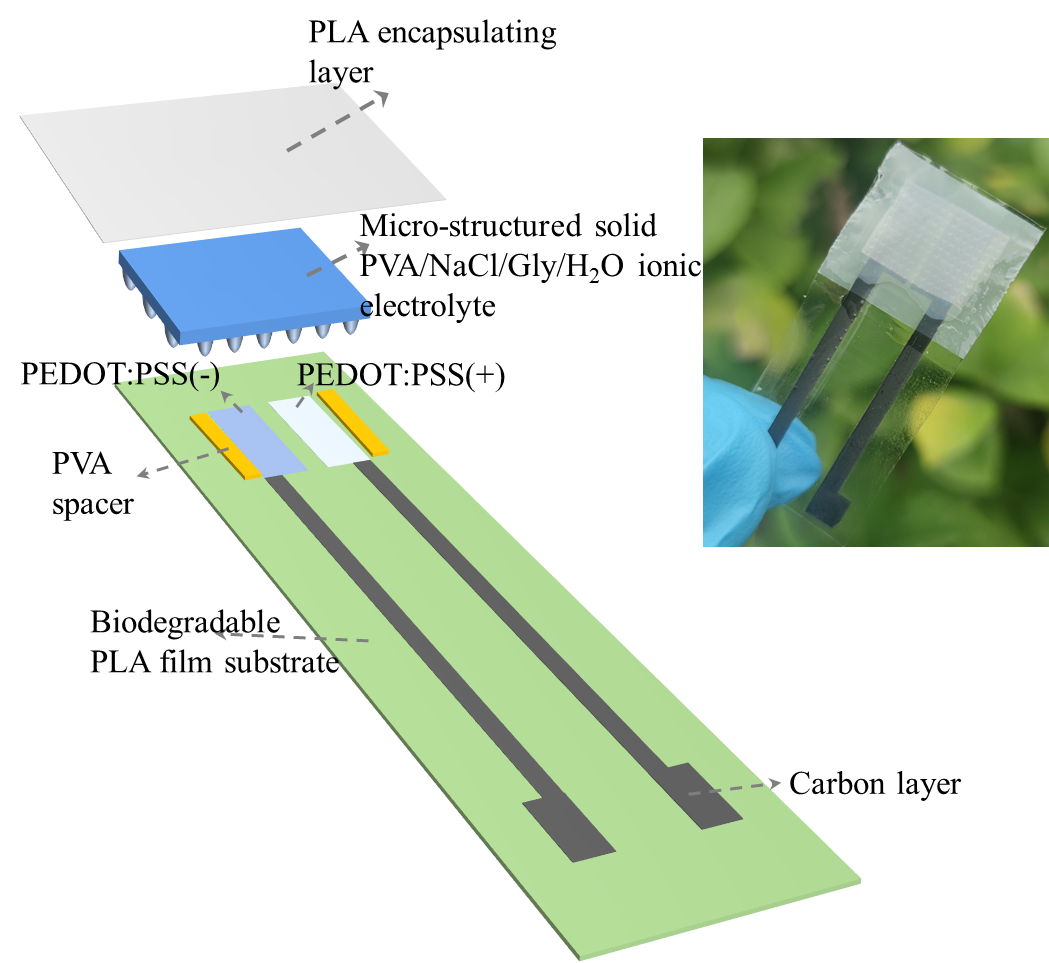


**Fig. S23** Schematic illustration and digital photograph of the proposed passive tactile sensors


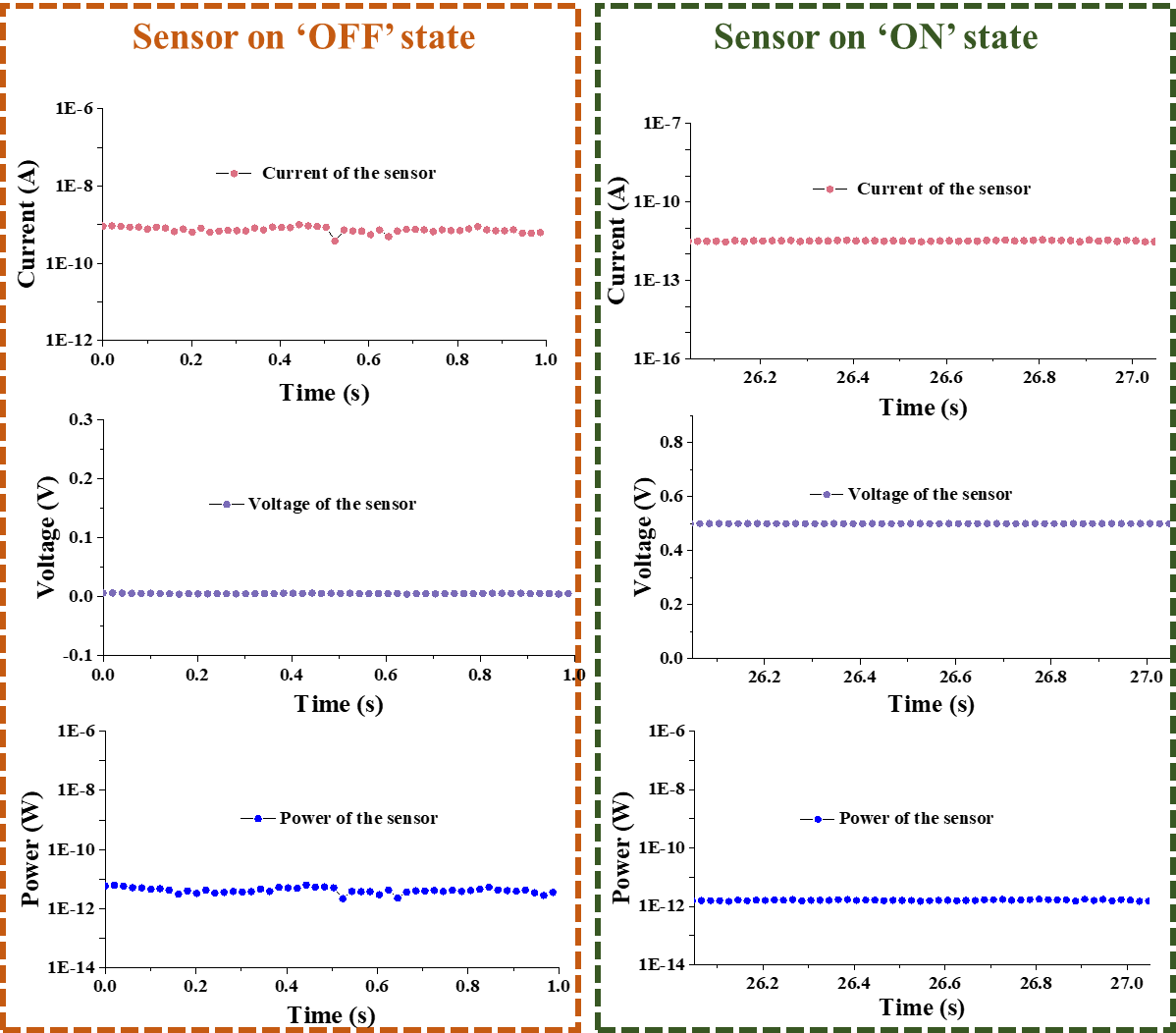


**Fig. S24** Recorded current, voltage, and overall power consumption of the sensor during the potentiometric measurement when the sensor is on ‘OFF’ and ‘ON’ states respectively


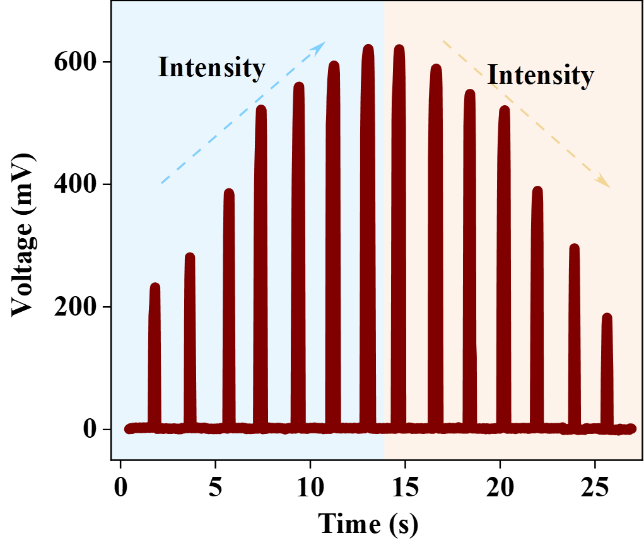


**Fig. S25** Signal outputs of the sensors with mechanical forces of different intensities applied upon the device


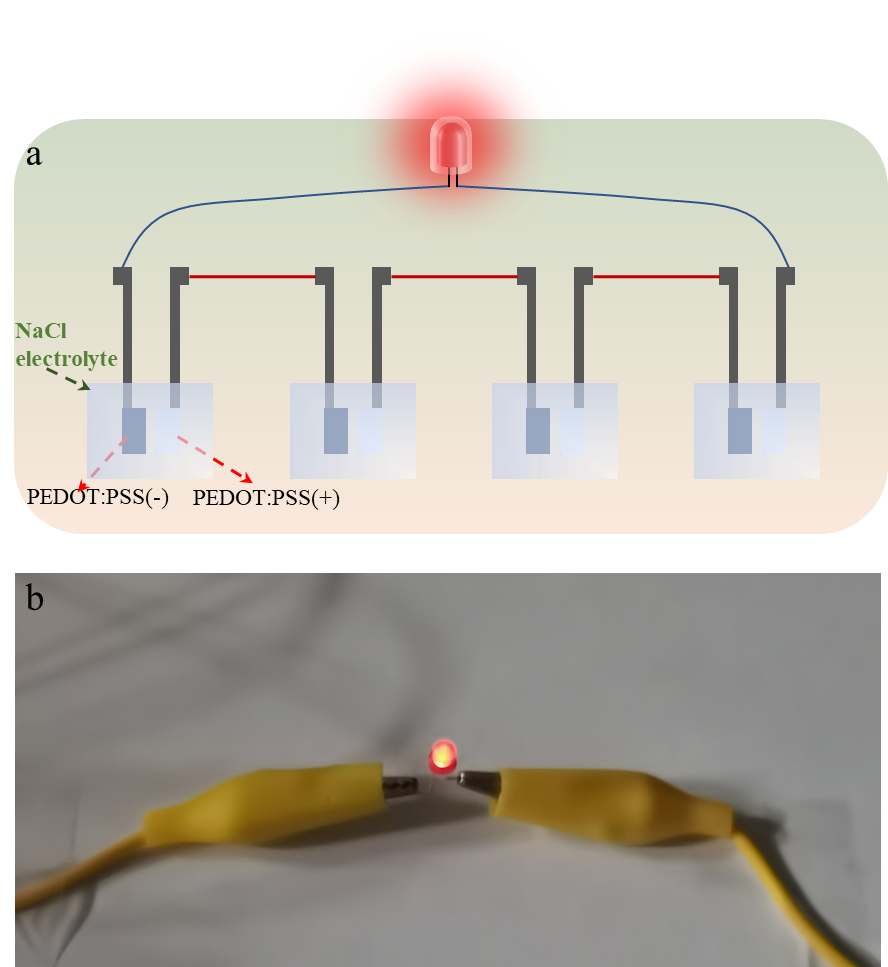


**Fig. S26** Schematic illustration (**a**) and photograph (**b**) showing that an LED light could be lighted up by four connected devices composed of PEDOT:PSS(+)/NaCl electrolyte (1 M)/PEDOT:PSS(-), demonstrating that the signal outputs of the tactile sensors are totally self-generated without external power supply


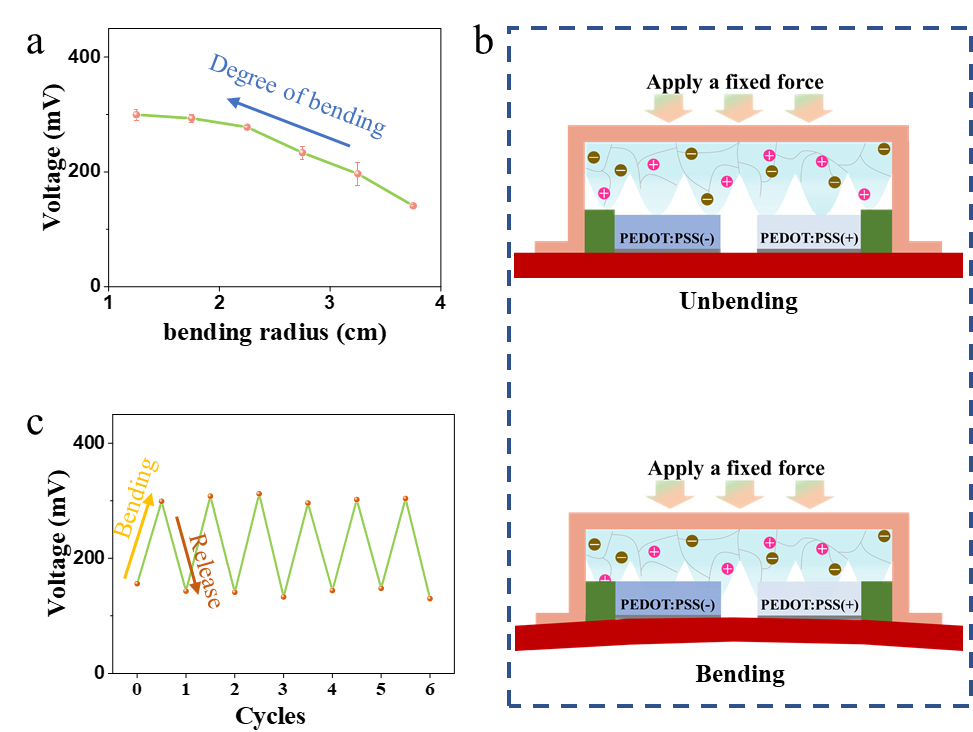


**Fig. S27** (**a**) Potential difference variations of the sensor under different bending radius. (**b**) Schematics illustrating the configurations of the tactile sensors before and after bending deformation. (**c**) The potential difference outputs measured at repeated bending and unbending processes for 6 cycles


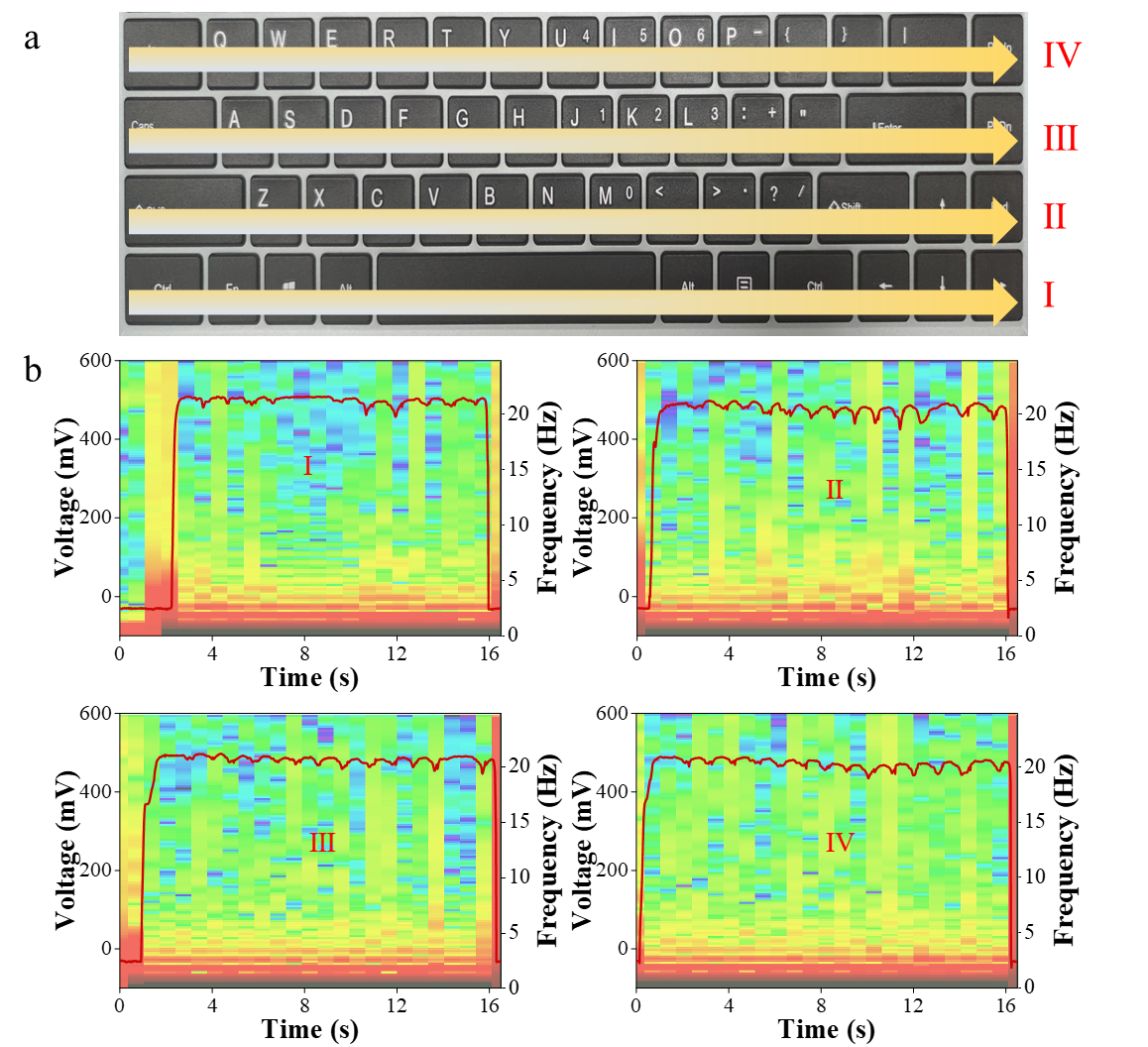


**Fig. S28** Photograph illustrating the four sliding paths on a keyboard (**a**). Recorded potential difference variations and the extracted STFT spectra when the fingertip equipped with the tactile sensors slides along path Ⅰ, Ⅱ, Ⅲ, and Ⅳ, respectively (**b**)


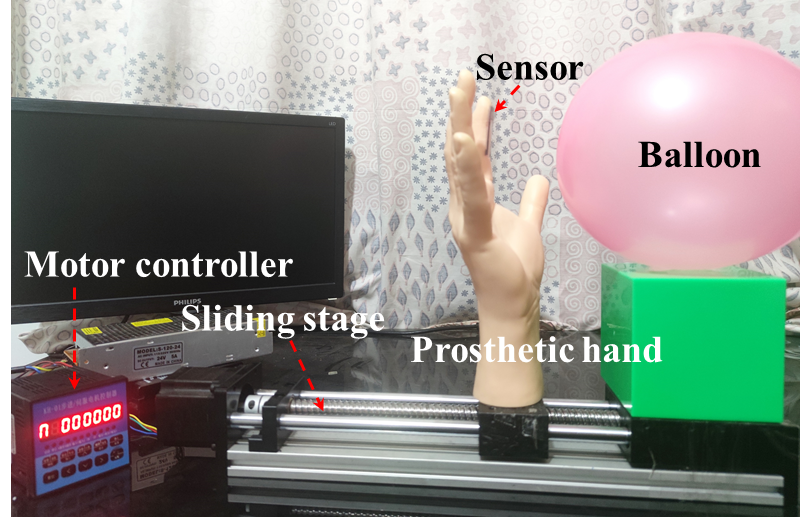


**Fig. S29** Photograph showing the setup used for detecting the material properties of different objects with a prosthetic hand equipped with the tactile sensors. The prosthetic hand equipped with the tactile sensors and the target objects were firmly fixed on a custom-made setup with a stepping motor, a motor controller, and a sliding stage. The moving distance, moving speed, and repeating cycles of the model hand to the objects can be digitally controlled. During this process, the potential difference variation signals of the tactile sensors are continuously recorded.


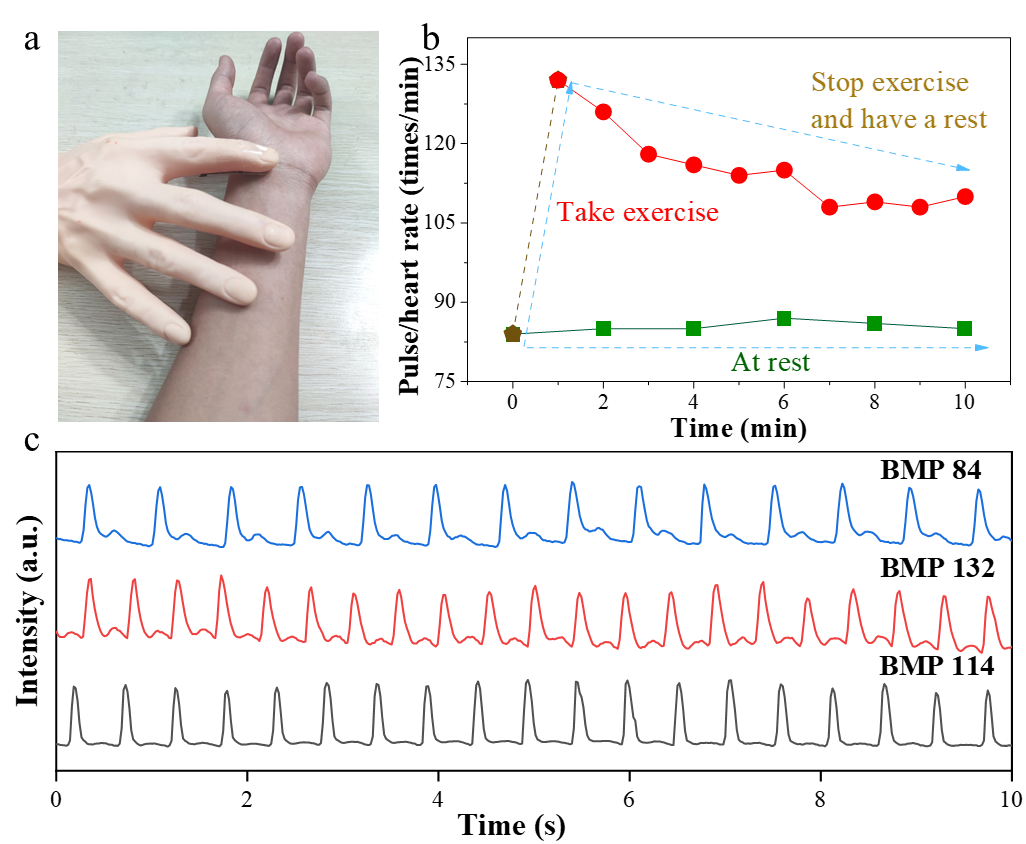


**Fig. S30** Photograph showing a prosthetic hand equipped with a tactile sensor placed on the wrist of a volunteer subject to detect the wrist pulse (**a**). The heart rate changes of the subject at resting state (green squares) and during taking exercise for 10 min (red circles) (**b**). Pulse signals recorded from the subject before taking exercise (top), after taking exercise (middle) and after resting for 5 min (bottom) (**c**)


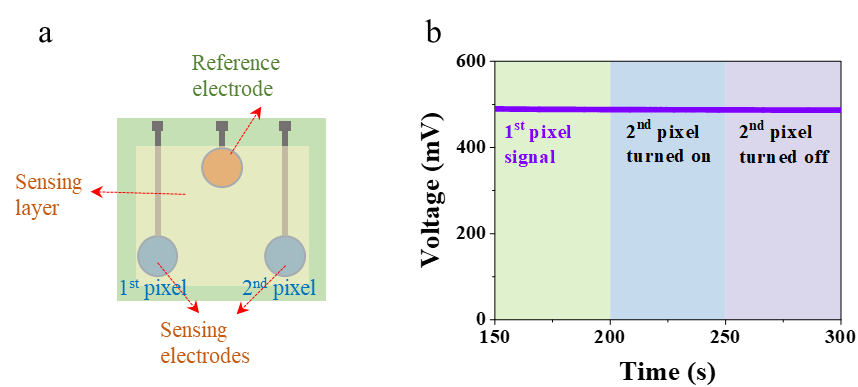


**Fig. S31** (**a**) Schematic showing a simplified tactile sensing array with one reference electrode, two sensing electrodes, and a sensing layer placed on top of the electrodes. The signals of the two sensing electrodes are measured simultaneously with respect to the reference electrode using two sets of equipment. Each sensing pixel is operated by applying and removing an external force. Firstly, the 1st pixel is turned on and measured continuously. Then, the 2nd pixel is turned on or off to observe the interference of the 2nd pixel to the signal of the 1st pixel. (**b**) Potential difference variation of the 1st pixel when the 2nd pixel is turned on or turned off. The two pixels working simultaneously will not affect each other and the mutual interference is minimal. This is because that, for the potential difference signal measurement, there is nearly no current flow involved in the nearly open-circuit potentiometric signal measurement. This enables the tactile sensing array to mitigate the issue of mutual interference between signals from different sensing pixels and wires


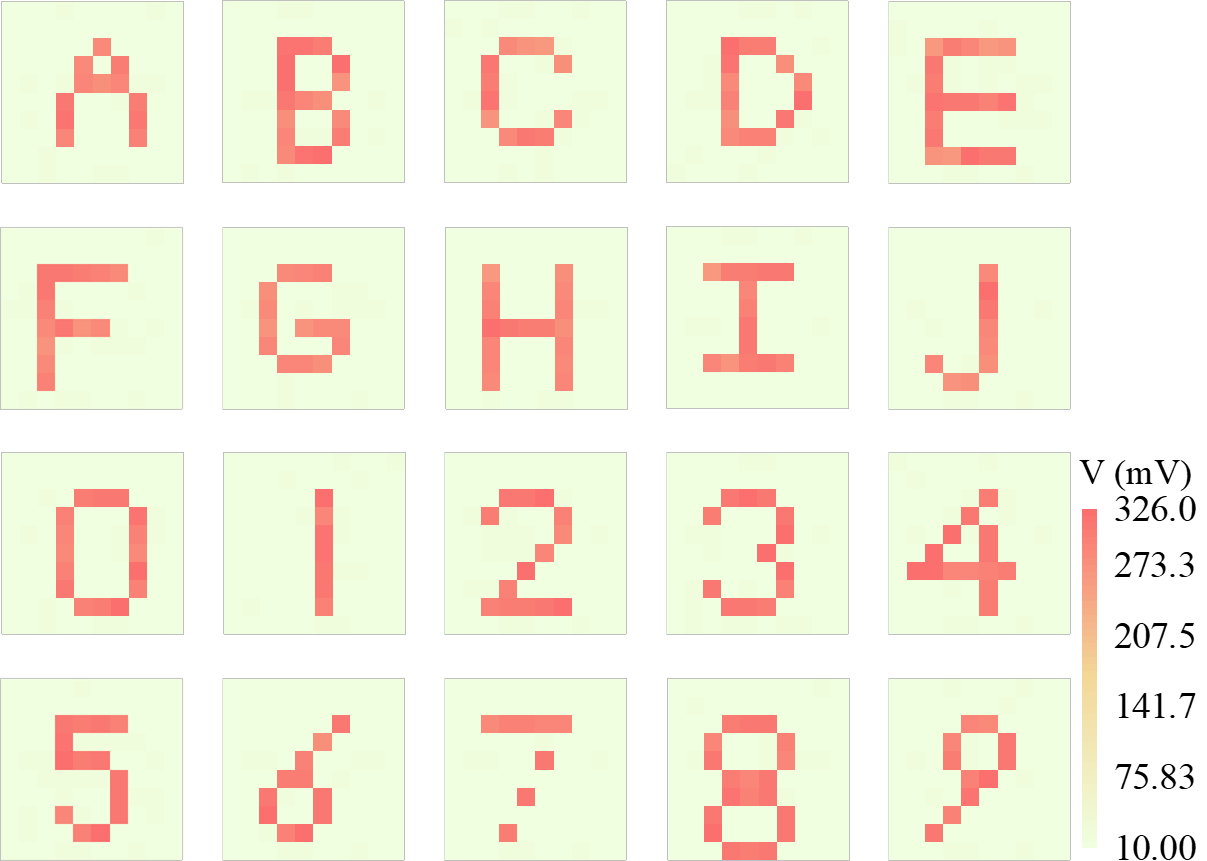


**Fig. S32** Spatial mappings in the potential difference outputs of the passive and single-electrode-mode e-skin when 3D printed plastic molds with different shapes are pressed onto the e-skin


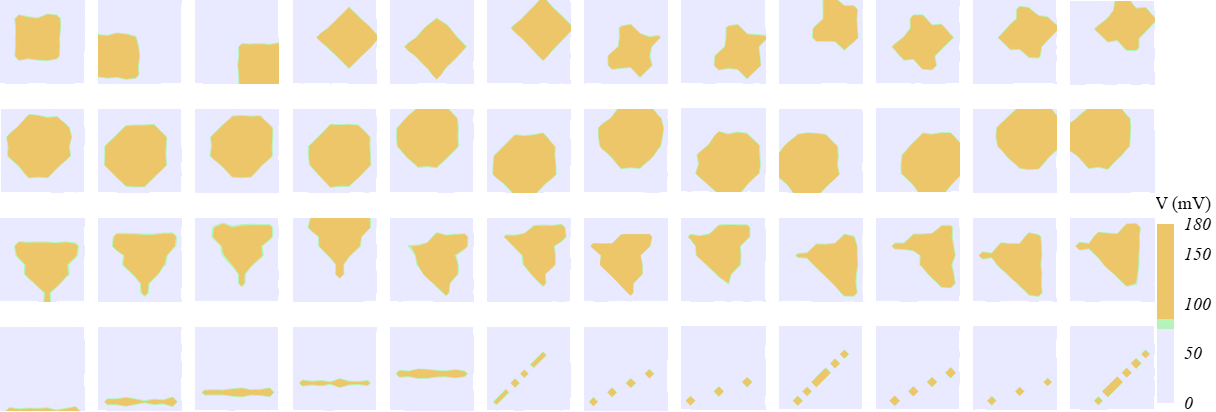


**Fig. S33** Reconstructed color mapping images when objects with different shapes (i.e., cube, cylinder, triangular prism, and rod) are placed to the e-skin at different positions and in different angles


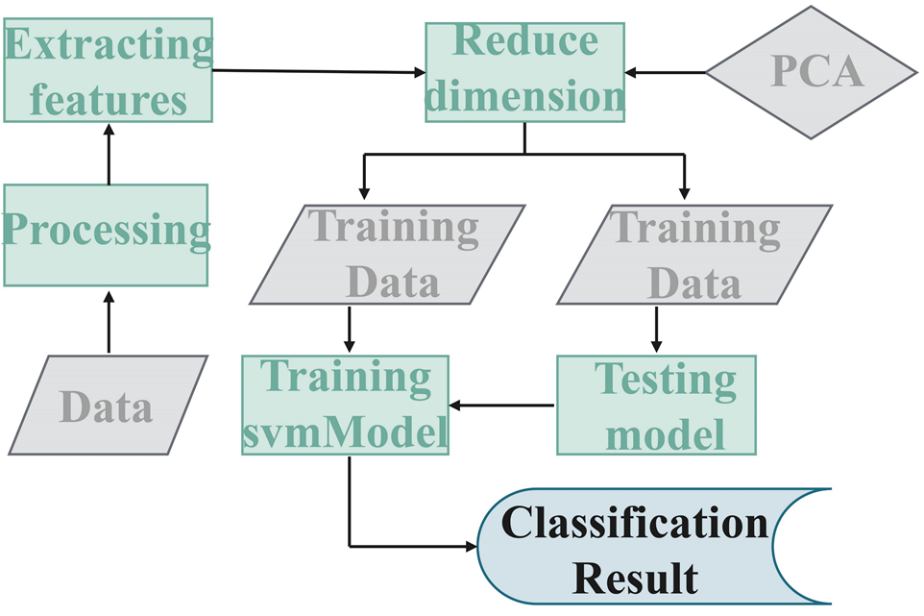


**Fig. S34** Diagram illustrating the structure of the image-based machine learning framework

**Supplementary References**

1. J. Rivnay, S. Inal, B.A. Collins, M. Sessolo, E. Stavrinidou et al., Structural control of mixed ionic and electronic transport in conducting polymers. Nat. Commun. **7**, 11287 (2016). <https://doi.org/10.1038/ncomms11287>
2. A.V. Volkov, K. Wijeratne, E. Mitraka, U. Ail, D. Zhao et al., Understanding the capacitance of pedot: pss. Adv. Funct. Mater. **27**, 1700329 (2017). <https://doi.org/10.1002/adfm.201700329>
3. M. Berggren, G.G. Malliaras, How conducting polymer electrodes operate. Science **364**, 233-234 (2019). <https://doi.org/10.1126/science.aaw929>
4. G. Rebetez, O. Bardagot, J. Affolter, J. Réhault, N. Banerji, What drives the kinetics and doping level in the electrochemical reactions of pedot: pss? Adv. Funct. Mater. **32**, 2105821 (2022). <https://doi.org/10.1002/adfm.202105821>
